# Distinct Ring1b complexes defined by DEAD-box helicases and EMT transcription factors synergistically enhance E-cadherin silencing in breast cancer

**DOI:** 10.1038/s41419-021-03491-4

**Published:** 2021-02-19

**Authors:** Yawei Wang, Yingying Sun, Chao Shang, Lili Chen, Hongyu Chen, Dake Wang, Xianlu Zeng

**Affiliations:** The Key Laboratory of Molecular Epigenetics of the Ministry of Education, Institute of Genetics and Cytology, Northeast Normal University, Changchun, Jilin China

**Keywords:** Breast cancer, Metastasis, Cadherins, Epithelial-mesenchymal transition

## Abstract

Ring1b is a core subunit of polycomb repressive complex 1 (PRC1) and is essential in several high-risk cancers. However, the epigenetic mechanism of Ring1b underlying breast cancer malignancy is poorly understood. In this study, we showed increased expression of Ring1b promoted metastasis by weakening cell–cell adhesions of breast cancer cells. We confirmed that Ring1b could downregulate E-cadherin and contributed to an epigenetic rewiring via PRC1-dependent function by forming distinct complexes with DEAD-box RNA helicases (DDXs) or epithelial-mesenchymal transition transcription factors (EMT TFs) on site-specific loci of E-cadherin promoter. DDXs-Ring1b complexes moderately inhibited E-cadherin, which resulted in an early hybrid EMT state of epithelial cells, and EMT TFs-Ring1b complexes cooperated with DDXs-Ring1b complexes to further repress E-cadherin in mesenchymal-like cancer cells. Clinically, high expression of Ring1b with DDXs or EMT TFs predicted low levels of E-cadherin, metastatic behavior, and poor prognosis. These findings provide an epigenetic regulation mechanism of Ring1b complexes in E-cadherin expression. Ring1b complexes may be potential therapeutic targets and biomarkers for diagnosis and prognosis in invasion breast cancer.

## Introduction

Breast cancer is the second leading cause of cancer death in women, and metastasis is widely accepted as the major reason for cancer mortality^1,2^. Cells from primary tumors gain an invasive phenotype through epithelial-mesenchymal transition (EMT)^3,4^. EMT is associated with the loss of cell–cell adhesions and downregulation of epithelial genes^5,6^. The silencing of E-cadherin enables cells to break loose from each other and is considered as a crucial step in EMT^7,8^. Multiple transcription factors are required for the transcriptional suppression of E-cadherin. EMT transcription factors (EMT TFs), such as Snail, Twist, and ZEB, are classic E-cadherin repressors exerting their effects on the proximal region of the E-cadherin promoter^9–14^. Noncanonical repressor DEAD-box RNA helicases (DDXs), such as DDX3X and DDX5, are also associated with the loss of E-cadherin in cancers, but DDX3X prefers to bind distal region of the E-cadherin promoter^15,16^. At present, it is not well understood how these factors couple to transcriptional repressors, and whether there are communications among these complexes on the E-cadherin promoter in cancer.

Polycomb group (PcG) proteins are transcriptional repressors that epigenetically modify chromatin and regulate chromatin structure to decide cell fate^17,18^. The monoubiquitination of Lys119 on histone H2A (H2AK119ub) and the trimethylation of Lys27 on histone H3 (H3K27me3) are, respectively, modified products of polycomb repressive complex 1/2 (PRC1/2)^18,19^. Ring1b is a core component of PRC1 and represses PRC1 targets expression by E3 ubiquitin ligase activity^20–22^. In stem and noncancerous cells, Ring1b is crucial for maintenance of stemness and differentiation^23,24^. However, there is an increased evidence that Ring1b is overexpressed in cancers and promotes oncogenic cell transformation^17,25–27^. These findings suggest that prevention of Ring1b’s abnormal expression and distribution in chromatin may inhibit tumorigenesis. Ring1b and PRC1 lack inherent DNA-specific binding activity, thus additional binding factors are necessary^28–30^. Therefore, it remains poorly understood how DDXs and EMT TFs recruit Ring1b on the E-cadherin promoter in breast cancer.

In this study, we sought to gain an insight into the function of Ring1b by elucidating its activation, protein interactome, and chromatin recruitment in human normal mammary and breast cancer cell lines. We have found that DDXs (DDX3X/DDX5) or EMT TFs (Snail1/Twist2) selectively decided the recruitment and distribution of Ring1b complexes on site-specific loci of E-cadherin promoter in various EMT states cells. In addition, clinical evidence showed that high expression of Ring1b with DDXs or EMT TFs predicted low levels of E-cadherin, metastatic behavior and poor prognosis. Our findings raise the possibility that targeting Ring1b complexes may provide a means of eliminating metastasis in breast cancer.

## Results

### Ring1b promotes EMT via PRC1-dependent function in breast cancer

**Fig. 1:**
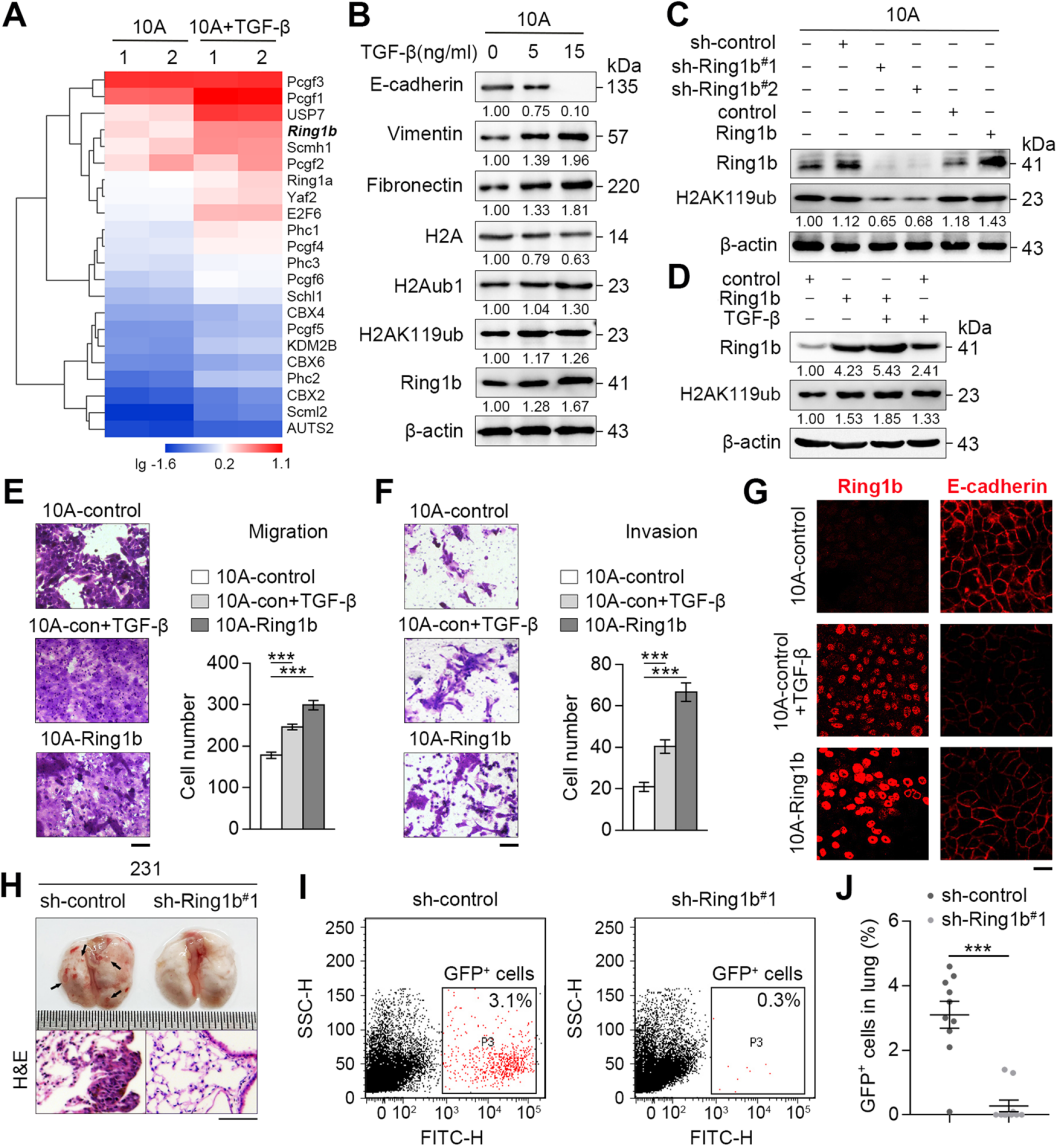
Ring1b is associated with breast cells metastasis in vitro and in vivo. Ring1b was stably overexpressed or knocked down in cells using lentivirus. **A** Heatmap of PRC1-genes expression influenced by TGF-β. Total RNAs isolated from 10 A and TGF-β-induced (15 ng/ml, 24 h) 10 A cells were analyzed by qRT-PCR. **B** Effect of TGF-β on H2Aub1, H2AK119ub, and Ring1b expression. Total protein extractions from 10 A, TGF-β-induced (24 h) 10 A cells were analyzed by western blot. **C** Effect of Ring1b on H2AK119ub expression. Total protein extractions were analyzed by western blot. **D** Effect of TGF-β and Ring1b on H2AK119ub expression. Total protein extractions from 10 A and TGF-β-induced (15 ng/ml, 24 h) 10 A cells were analyzed by western blot. **E, F** Function of Ring1b in cell migration and invasion. 10 A cells were stimulated with TGF-β (15 ng/ml) for 24 h. Statistical analysis of cells migration and invasion by Transwell assays are presented in the bar graphs. Scale bar, 60 μm. **G** Immunofluorescent staining of Ring1b (red; TRITC) and E-cadherin (red; TRITC) in 10 A cells. 10 A cells were stimulated with TGF-β (15 ng/ml) for 24 h. Scale bar, 25 μm. **H** Bright field images and H&E staining of lungs at 6 weeks after mouse tail vein injection. Scale bar, 75 μm. **I, J** Flow cytometry analysis of GFP^+^ 231 cells in lungs at 6 weeks after tail vein injection. Statistical analysis shows the percentage of GFP^+^ 231 cells in the lung. Error bars represent the means ± SEM. Unpaired *t*-test is performed to indicate a statistically significant difference. ***P < 0.001.

TGF-β-induced EMT models have been reported in multiple cancers^6^. To determine whether Ring1b-dependent epigenetic remodeling occurs in EMT, we treated breast normal epithelial cells MCF-10A (10 A) with TGF-β, and examined the expression of PRC1-genes using qRT-PCR arrays. The data from two independent experiments showed that expression of most PRC1-genes was upregulated in EMT (Fig. 1a). Further experiments confirmed that expressions of Ring1b and some canonical PRC1-genes were enhanced in TGF-β-induced 10 A cells, and high metastatic breast cancer cells MDA-MB-231 (ref. ^25^) (231) also expressed high levels of Ring1b (Fig. S1A). Western blot analysis showed that both Ring1b and its modified product H2AK119ub were increased under EMT state (Fig. 1b). To further investigate the key role of Ring1b-dependent epigenetic remodeling during EMT, expression of Ring1b was altered by lentivirus in different breast cell lines. Western blot data showed that expression of H2AK119ub was Ring1b-dependent in 10 A, 231 and epithelial-like cancer cells MCF-7, which are less metastatic than 231 cells^25,31^ (Fig. 1c and d; Fig. S1B and C). These results suggested that Ring1b and its dependent epigenetic remodeling are strengthened in EMT.

Next, we determined the function of Ring1b in cancer metastasis in vitro and in vivo. Using transwell assay, we found that overexpressing Ring1b resulted in an increase in migration and invasion in 10 A cells (Fig. 1e and f), and knockdown of Ring1b led to a decrease in migration and invasion in 231 cells (Fig. S1D and E). Using immunofluorescence assay, we observed that Ring1b showed an inhibitory effect on E-cadherin expression, suggesting an initiative role of Ring1b in EMT (Fig. 1g; Fig. S1F). To confirm our results in a more physiological environment, we injected GFP^+^ 231 cells into mouse tail vein (Fig. S1G). Six weeks after the injection, mice in the sh-Ring1b group gained more weight and had fewer metastatic nodules in the lungs (Fig. 1h; Fig. S1H). Flow cytometry analysis of GFP^+^ sorted cells showed that sh-Ring1b alleviated the metastatic capacity of cancer cells in the lungs (Fig. 1i and j). In addition, we explored the effect of Ring1b on proliferation, and found that Ring1b did not obviously affect cell cycle and viability within 48 h (Fig. S2A–D).

Taken together, these findings suggest that Ring1b helps cells evolve to a more aggressive phenotype via PRC1-dependent function in vitro and in vivo.

### DDXs and EMT TFs selectively recruit Ring1b complexes in different breast cell lines

**Fig. 2:**
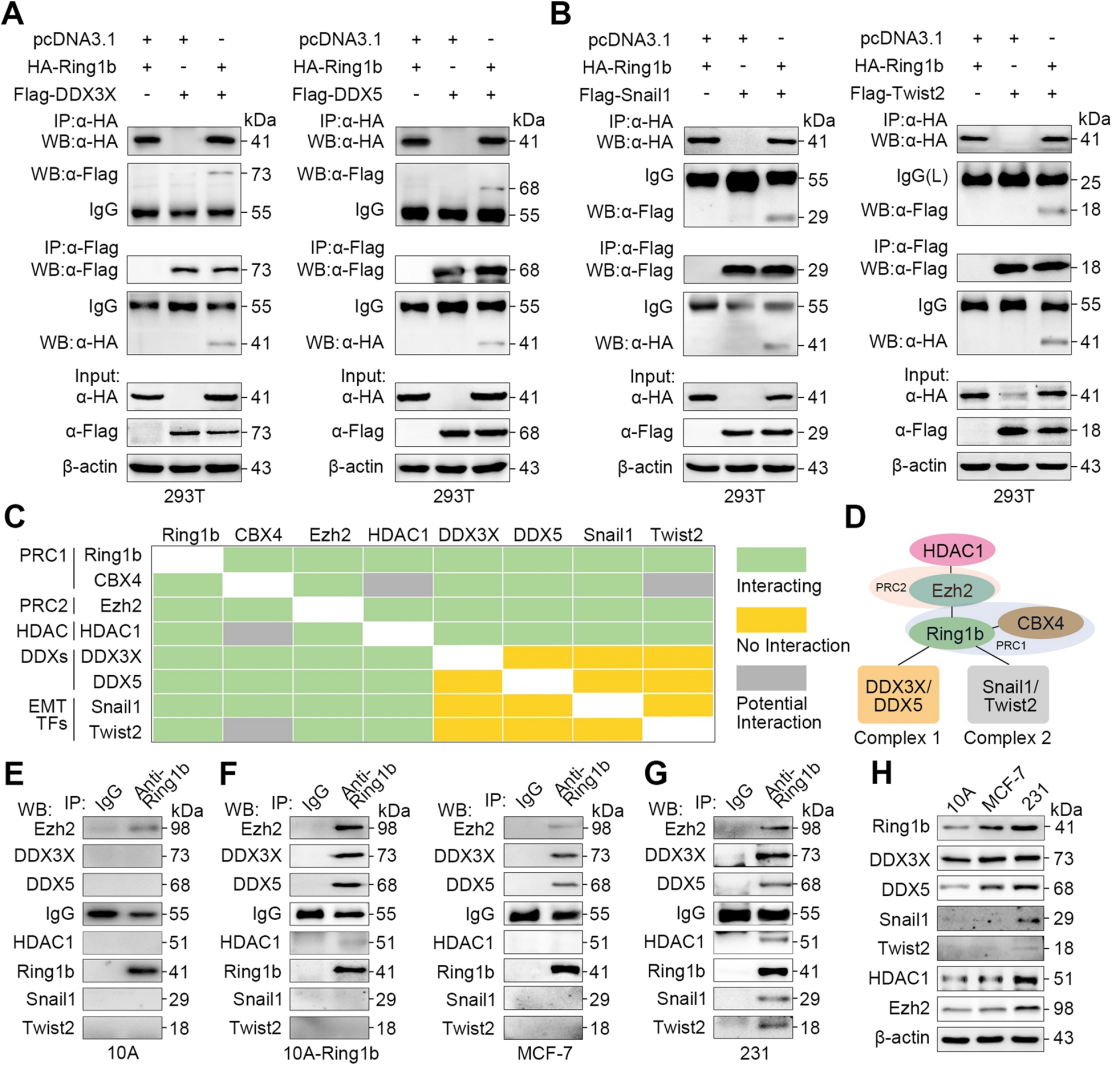
Verification of Ring1b-interacting proteins. **A, B** Ring1b interacts with DDX3X and DDX5, or with Snail1 and Twist2. The 293 T cells were transfected with plasmids as indicated for 24 h. Proteins were captured by IP and analyzed by western blot. **C** Heatmap of interaction between Ring1b-associated proteins. **D** Schematic depiction of Ring1b complexes. **E**–**G** Ring1b complexes verified in breast cell lines. Lysates from 10 A, 10A-Ring1b, MCF-7, and 231 cells were analyzed by IP followed by western blot. **H** Expression of endogenous Ring1b-associated proteins in breast cell lines. Total protein extractions from cells were analyzed by western blot.

Additional binding factors are necessary to help Ring1b recruit to chromodomains^28–30^. Therefore, cellular extracts from multiple breast cell lines were analyzed by immunoprecipitation (IP), SDS-PAGE, silver staining and mass spectrometry assays to capture proteins that interacted with Ring1b. Preliminary results showed that DDX3X, and DDX5 proteins were found in all samples, but Snail1 and Twist2 proteins were only found in 231 samples (Fig. S3A). DDXs and EMT TFs have been reported as E-cadherin repressors^6,15,16^, so we selected these proteins for a further research in metastasis. In addition, HDAC1 and other polycomb proteins Ezh2 and CBX4 (Fig. S4) were selected for further analysis of the composition of Ring1b complexes. Co-IP and western blot showed that exogenous DDX3X, DDX5, Snail1, and Twist2 co-purified with Ring1b, and all these proteins co-purified with Ezh2 and HDAC1 in 293 T cells (Fig. 2a and b; Fig. S3B–F). DDX3X/DDX5 did not interact with Snail1/Twist2 (Fig. S3G), suggesting that DDXs and EMT TFs may form distinct Ring1b complexes. We created a heatmap of Ring1b-associated proteins according to protein interaction analysis (Fig. 2c), and defined two Ring1b complexes, termed Complex 1 and 2 for convenience (Fig. 2d).

Next, the presence of Complex 1 and 2 were confirmed in breast cell lines. In 10 A cells, only Ezh2 interacted with Ring1b, suggesting no formation of Complex 1 and 2 (Fig. 2e). In 10A-Ring1b and MCF-7 cells, Ring1b interacted with DDX3X/DDX5, but did not bind Snail1/Twist2 (Fig. 2f; Fig. S4A and B), suggesting that Complex 2 does not exist in these cells, and HDAC1 was not captured by Ring1b, DDX3X and DDX5 in 10A-Ring1b and MCF-7 cells, suggesting that it’s an optional unit in complexes. In 231 cells, we detected both Complex 1 and 2 (Fig. 2g; Fig. S4C) (Endogenous protein expressions are shown in Fig. 2h). These data suggest that DDXs and EMT TFs selectively recruit Ring1b complexes in different breast cell lines.

### Ring1b complexes defined by DDXs or EMT TFs function as E-cadherin repressors

**Fig. 3:**
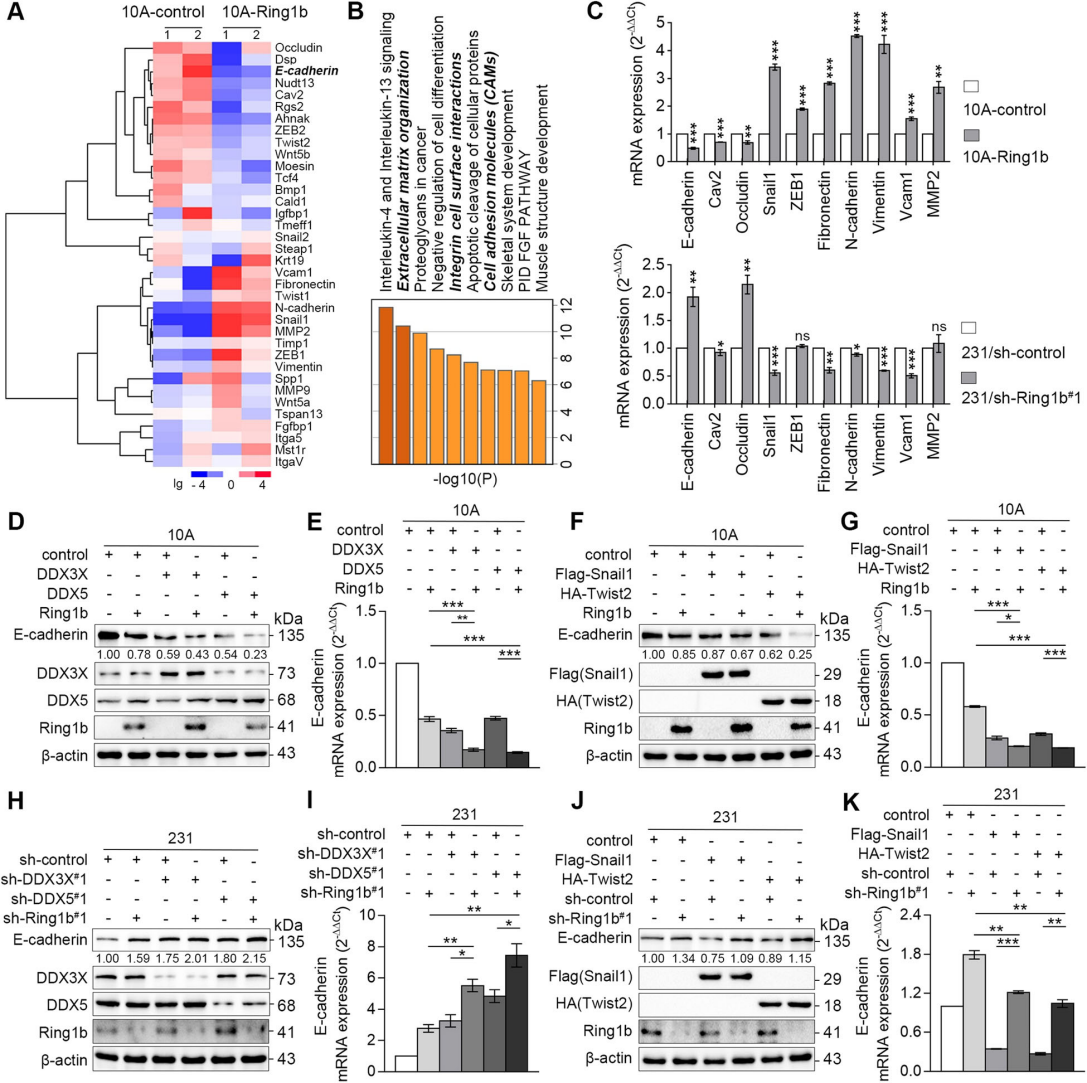
Ring1b cooperates with DDXs or EMT TFs to strengthen the inhibition of E-cadherin. Proteins as indicated were stably overexpressed or knocked down in cells using lentivirus. **A** Clustering analysis of EMT-related genes’ expression influenced by Ring1b. Total RNAs isolated from 10A-control and 10A-Ring1b cells were analyzed by qRT-PCR. **B** Gene Ontology (GO) term analysis of EMT-related genes influenced by Ring1b. **C** Effect of Ring1b on epithelial and epithelial genes expression. Total RNAs isolated from 10 A and 231 cells were analyzed by qRT-PCR. **D–K** Effect of Ring1b complexes on E-cadherin expression in 10 A and 231 cells. Total protein and RNAs isolated from cells were analyzed for E-cadherin expression by western blot and qRT-PCR. Error bars represent the means ± SEM. Unpaired *t*-test is performed to indicate a statistically significant difference. ns, *P* ≥ 0.05; *P < 0.05; **P < 0.01; ***P < 0.001.

To explore target genes of Ring1b complexes in breast cancer cells, we determined expressions of EMT-genes in 10 A cells by qRT-PCR arrays. The data from two independent experiments showed that overexpression of Ring1b in 10 A cells down-regulated 54% of epithelial genes (≤0.75-fold) and upregulated 52% of mesenchymal genes (≥1.25-fold) (Fig. 3a). Gene Ontology (GO) term analysis indicated that these genes are mainly involved with cell adhesion and extracellular matrix organization (Fig. 3b). We selected genes that are associated with cell adhesion for further investigation. The results showed that most of these genes, including E-cadherin, might be targets of Ring1b in 10 A and 231 cells (Fig. 3c).

**Fig. 4:**
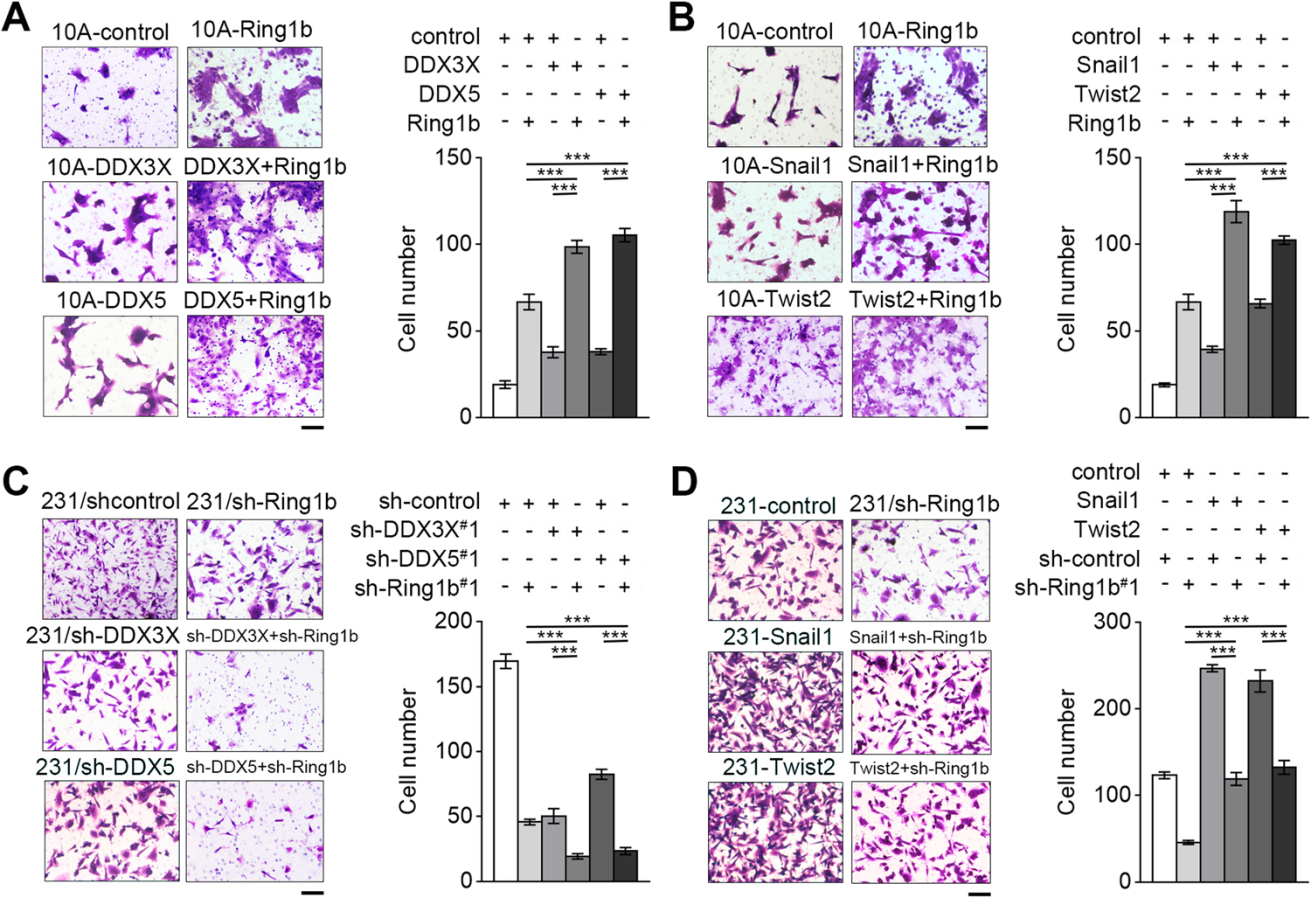
Ring1b complexes promote invasion in breast cells. Proteins as indicated were stably overexpressed or knocked down in cells using lentivirus. **A–D** Effect of Ring1b complexes on cell invasion in 10 A and 231 cells. Statistical analysis for cells migration and invasion by Transwell assays are presented in the bar graphs. Scale bar, 60 μm. Error bars represent the means ± SEM. Unpaired *t*-test is performed to indicate a statistically significant difference. ***P < 0.001.

DDXs and EMT TFs have been reported as E-cadherin repressors^6,15,16^. Thus, we hypothesized that Ring1b may cooperate with DDXs and EMT TFs to silence E-cadherin in breast cancer. To determine whether this is the case, we, respectively, altered DDX3X, DDX5, Snail1, and Twist2 in 10 A and 231 cells, and confirmed their effects on E-cadherin expression and metastasis (Fig. S5A–L; Fig. S6A–F). Importantly, co-overexpression of Ring1b with DDX3X/DDX5 or Snail1/Twist2 in 10 A cells resulted in lower expression of E-cadherin (Fig. 3d–g), and knockdown of Ring1b and DDX3X/DDX5 in 231 cells further increased the expression of E-cadherin (Fig. 3h and i). In addition, overexpression of Snail1/Twist2 in 231/sh-Ring1b cells did not fully rescue the inhibition of E-cadherin, indicating the essential role of Ring1b in the function of EMT TFs (Fig. 3j and k). Transwell invasion analysis showed that DDXs or EMT TFs could further enhance cell invasion by cooperating with Ring1b (Fig. 4a–d). Taken together, our results suggest that DDXs and EMT TFs function as co-repressors with Ring1b on E-cadherin in breast cancer.

### DDXs and EMT TFs synergistically recruit of Ring1b complexes to the E-cadherin promoter

**Fig. 5:**
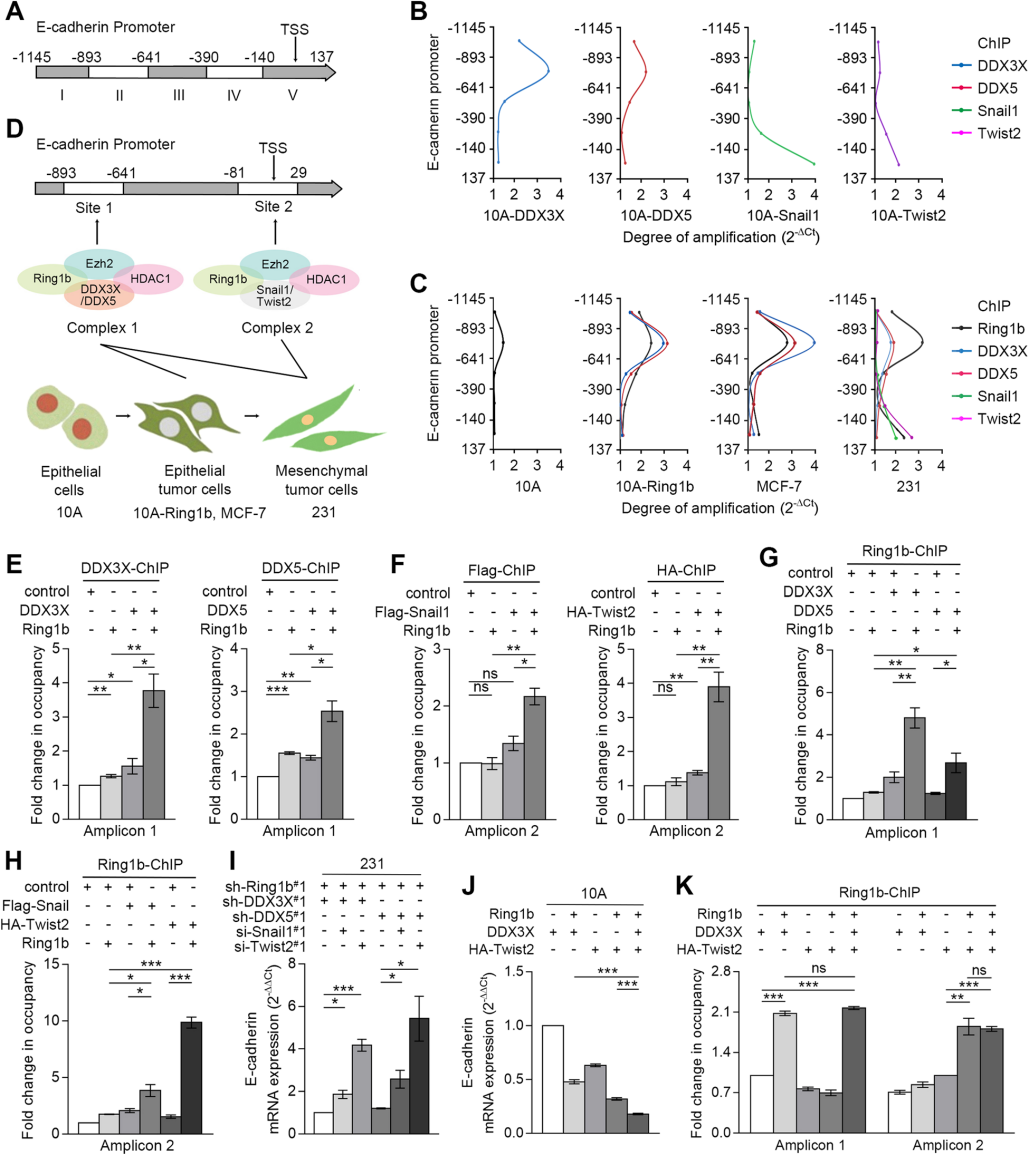
Ring1b complexes inhibit E-cadherin expression at transcriptional level. Proteins as indicated were stably overexpressed or knocked down in cells using lentivirus or si-RNA. IgG was used as control antibody. The probability of proteins binding to the E-cadherin promoter was analyzed by ChIP-qRT-PCR. **A** Schematic representation of E-cadherin promoter region. E-cadherin promoter was divided into five regions to determine the degree of amplification. **B, C** DNA binding ability of DDXs, EMT TFs, and Ring1b on the E-cadherin promoter. Immunoprecipitated DNA fragments were captured by antibodies specific to DDX3X, DDX5, Flag (Snail1), HA (Twist2), and Ring1b in breast cell lines. **D** Schematic diagram of Ring1b complexes associated with metastatic features in breast cells. **E–H** Effect of Ring1b complexes on E-cadherin transcription. Immunoprecipitated DNA fragments from 10 A cells were captured by antibodies specific to Ring1b, DDX3X, DDX5, Flag (Snail1), and HA (Twist2). **I, J** Effect of three proteins on E-cadherin expression. Total RNAs isolated from 231 and 10 A cells were analyzed by qRT-PCR. **K** Recruitment of Ring1b at site 1 and 2 in 10 A cells. Immunoprecipitated DNA fragments from 10 A cells were captured by antibody specific to Ring1b. Error bars represent the means ± SEM. Unpaired *t*-test is performed to indicate a statistically significant difference. ns, *P* ≥ 0.05; *P < 0.05; **P < 0.01; ***P < 0.001.

Previous studies have shown that DDXs and EMT TFs occupy different DNA binding sites of the E-cadherin promoter^9,15,32^. To assess the binding ability of Complex 1 and 2 on the E-cadherin promoter, we designed primer pairs to amplify the coeluted DNA fragment by ChIP assay (Fig. 5a). The results showed that DDX3X/DDX5 had high DNA binding ability on region II, and Snail1/Twist2 on region V (Fig. 5b). In 10 A, 10A-Ring1b and MCF-7 cells, Ring1b, DDX3X, and DDX5 only bound region II (Fig. 5c). In 231 cells, Ring1b bound both region II and V, DDX3X and DDX5 bound region II, and Snail1/Twist2 bound region V (Fig. 5c). These data suggest that DDXs and EMT TFs selectively recruit Ring1b complexes on site 1 or 2 in different breast cell lines (Fig. 5d).

Further research focused on the cooperation of Complex 1 and 2 in E-cadherin transcription. ChIP analysis showed that co-overexpression of Ring1b with DDX3X/DDX5 or Snail1/Twist2 resulted in the highest probability of these proteins to bind site 1 or 2 (Fig. 5e–h). To explore whether there was a competitive relationship between Complex 1 and 2, we constructed cell lines that altered three proteins. Results showed that co-knockdown or -overexpression of the three proteins together, respectively, achieved the highest or lowest expression of E-cadherin (Fig. 5i and j). ChIP analysis demonstrated that the efficiency of Ring1b in occupying two sites was not reduced, suggesting that Complex 1 and 2 has no competition on the E-cadherin promoter (Fig. 5k).

### Distinct Ring1b complexes facilitate rewiring of epigenetic markers on the E-cadherin promoter

**Fig. 6:**
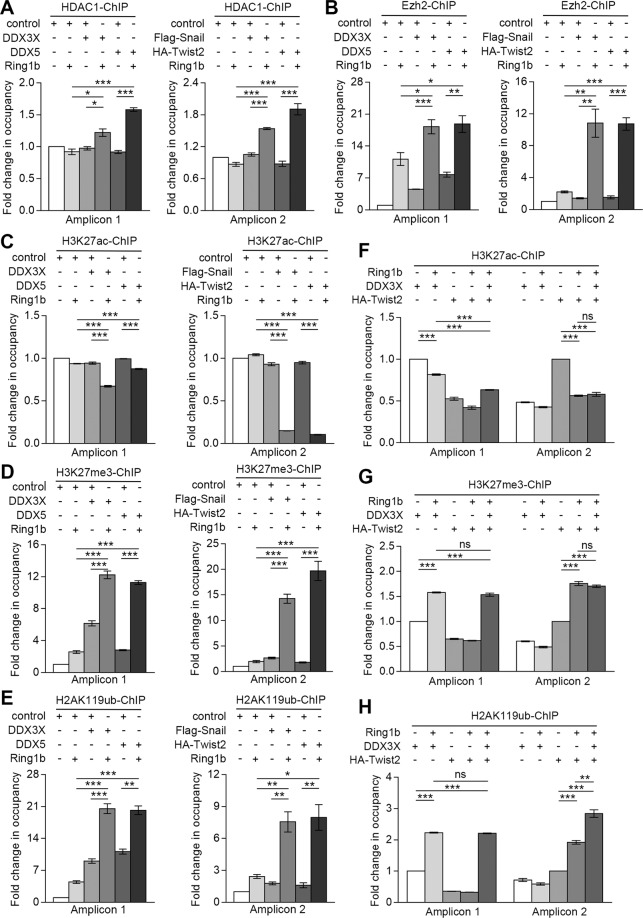
Ring1b-dependent epigenetic remodeling is associated with multiple epigenetic markers. Proteins as indicated were stably overexpressed or knocked down in 10 A cells using lentivirus. IgG was used as control antibody. Immunoprecipitated DNA fragments were captured by antibodies as indicated. The probability of proteins binding to E-cadherin promoter was analyzed by ChIP-qRT-PCR. **A, B** Effect of HDAC1 and Ezh2 on E-cadherin transcription. **C**–**E** Effect of Ring1b complexes on epigenetic markers distribution on the E-cadherin promoter. **F**–**H** Effect of three proteins on epigenetic markers distribution on the E-cadherin promoter. Error bars represent the means ± SEM. Unpaired *t*-test is performed to indicate a statistically significant difference. ns, *P* ≥ 0.05; *P < 0.05; **P < 0.01; ***P < 0.001.

To ascertain whether the transcription of E-cadherin was defined by the dynamic epigenetic alterations, we performed ChIP with antibodies in 10 A cells. Analysis showed that overexpression of Complex 1 or 2 resulted in the highest Ezh2, HDAC1, H3K27me3, and H2AK119ub, and the lowest H3K27ac (substrate of HDAC1) in occupancy of E-cadherin promoter (Fig. 6a–e). In addition, we found that co-expressing three proteins could not reduce the distributions of H3K27me3 and H2AK119ub, or increase the distributions of H3K27ac on the E-cadherin promoter compared with cells co-expressing two proteins, implying a noncompetitive relation between Complex 1 and 2 (Fig. 6f–h).

**Fig. 7:**
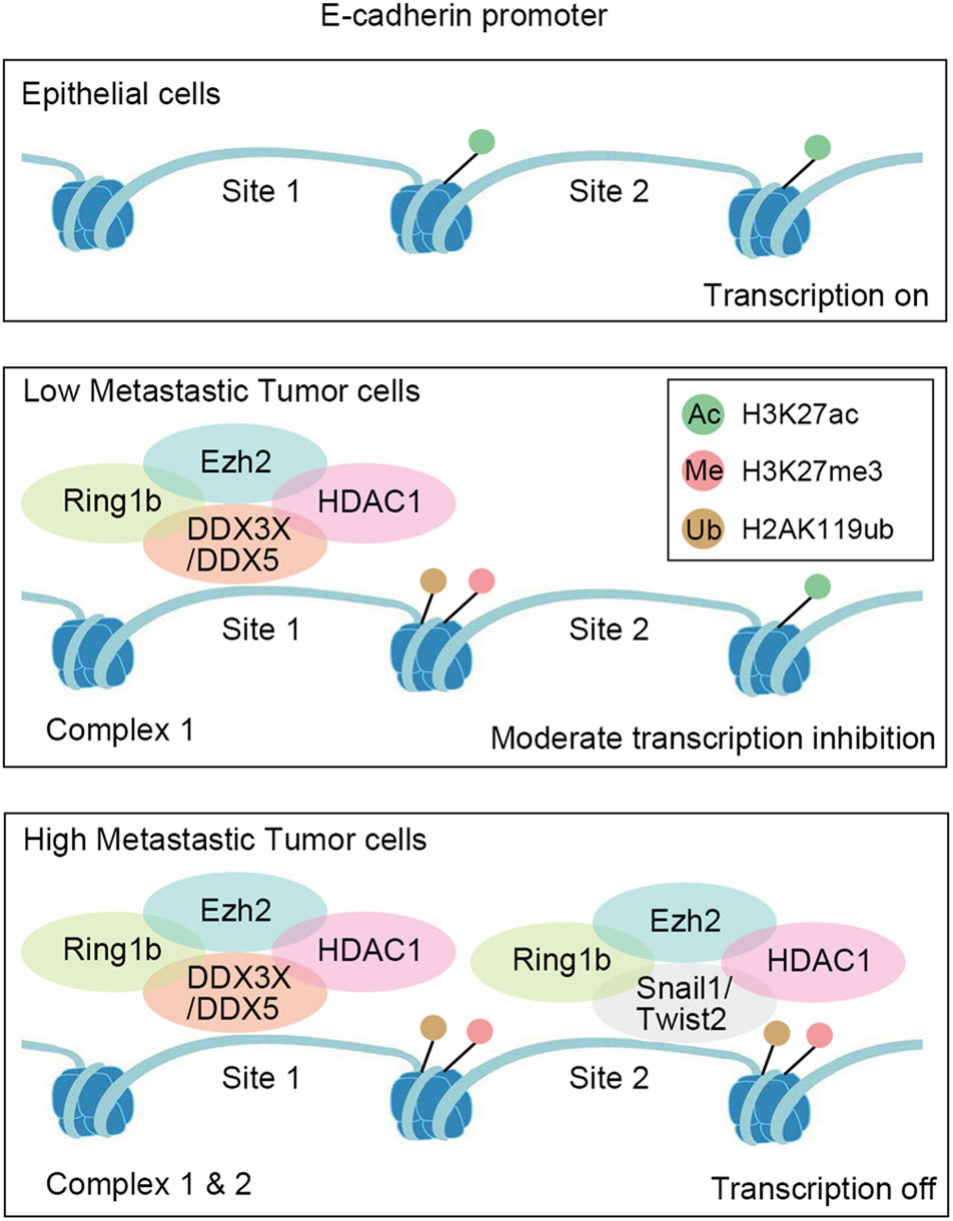
Schematic diagram of Ring1b-dependent epigenetic remodeling for E-cadherin transcription in breast cell lines. Distinct Ring1b complexes gradually make breast cells lose the expression of E-cadherin, change epigenetic markers on the E-cadherin promoter and acquire metastatic characteristics.

We infer that the transcription of E-cadherin is associated with alterations of epigenetic markers. In normal epithelial cells (10 A), site 1 and site 2 are not occupied by Ring1b complexes, resulting in the highest expression of H3K27ac and E-cadherin. In epithelial-like tumor cells or an early EMT state of epithelial cells (MCF-7 and 10A-Ring1b), site 1 is occupied by Complex 1, leading to alterations of epigenetic markers on distal region of the E-cadherin promoter and a moderate inhibition of E-cadherin. In mesenchymal-like cancer cells (231), site 1 and site 2 are, respectively, occupied by Complex 1 and Complex 2, leading to alterations of epigenetic markers on distal and proximal region of the E-cadherin promoter and the silencing of E-cadherin (Fig. 7).

### High levels of Ring1b with DDXs or EMT TFs predict metastasis and poor prognosis in patients with breast cancer

**Fig. 8:**
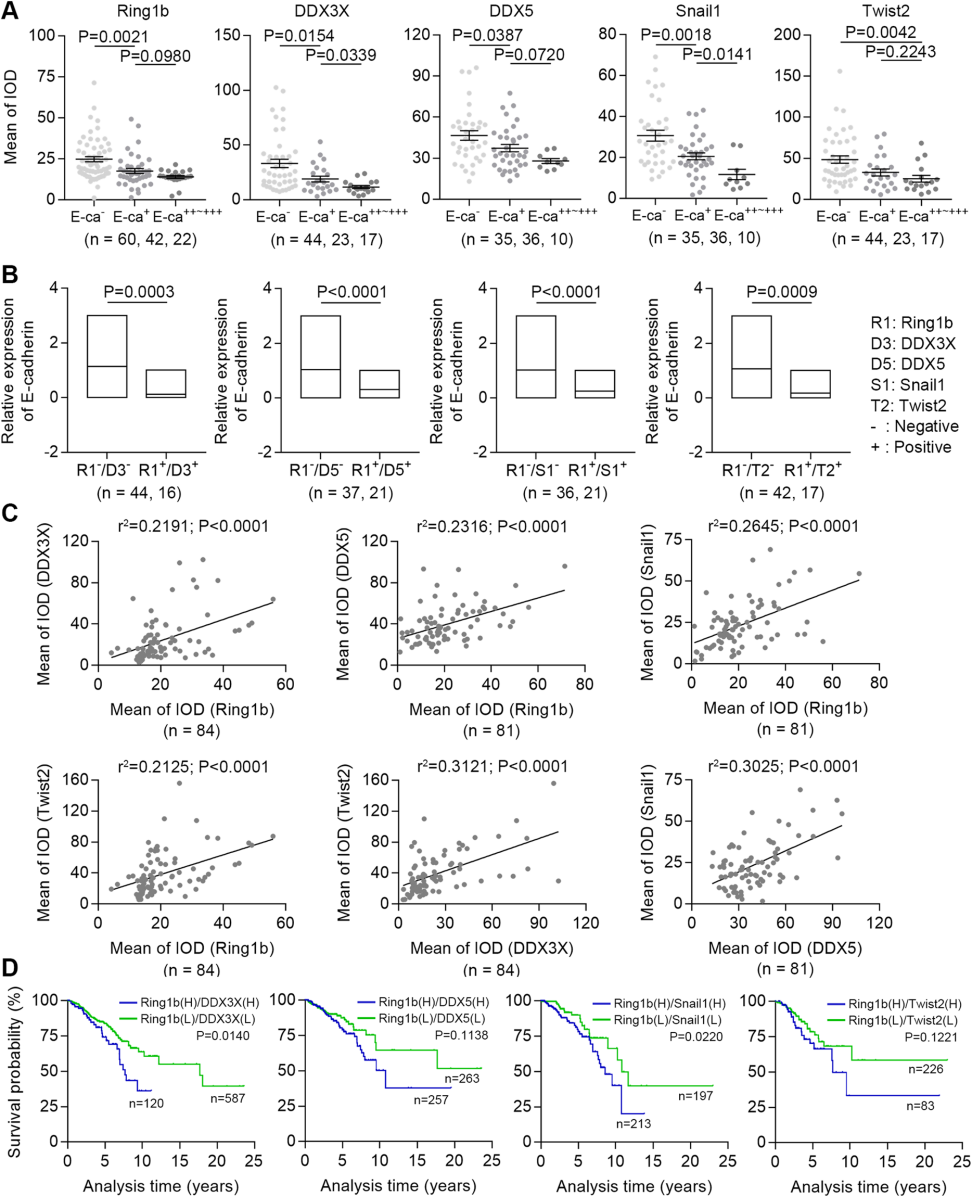
Ring1b and associated proteins predict breast cancer metastasis. Mean integrated optical density (IOD) was used to evaluate expression of proteins in tissues. E-cadherin expression was determined by immunoreactive scoring system (− negative, + weak, ++ to +++ moderate and strong, respectively). **A** Single factor analysis of E-cadherin expression in cancer tissues. **B** Double factor analysis of E-cadherin expression in cancer tissues. **C** Correlation analysis of Ring1b and associated proteins in cancer tissues. **D** Double factor analysis of survival curves in cancer tissues. The data from TCGA atlas were analyzed by Kaplan–Meier method. Paired *t*-test is performed to indicate a statistically significant difference.

We collected 37 samples of invasive ductal carcinoma and adjacent breast tissue (normal tissue), and 87 samples of invasive ductal carcinoma and adjacent lymph node tissue (metastatic cancer tissue) (Table. S1). Immunohistochemistry experiment results showed that the staining of Ring1b, DDX3X, DDX5, Snail1, and Twist2 in cancer tissues and metastatic cancer tissues was stronger compared with that in adjacent normal tissues (Fig. S7A). Statistical analysis showed that cancer tissues expressed high levels of these proteins compared with adjacent normal tissues, and metastatic cancer tissues showed the highest expression of these proteins compared with adjacent primary cancer tissues (Fig. S8A–E). High expression of these proteins all reflected low expression of E-cadherin in cancer tissues (Fig. 8a). Moreover, high levels of Ring1b with DDXs or EMT TFs, or DDXs with EMT TFs in cancer tissues predicted lower expression of E-cadherin (Fig. 8B; Fig S9A). In addition, we found that the expression of Ring1b was positively correlated with other proteins, indicating the key role of Ring1b in cancer metastasis (Fig. 8c). By the analysis of survival curve form database, we found that high expression of Ring1b/DDXs/EMT TFs predicted poorer prognosis comparing patients who highly expression just one of these factors, and Ring1b complexes were valuable predictors of survival in patients with breast cancer (Fig. 8d; Fig. S10A and B).

## Discussion

Previous studies have shown that Ring1b is an oncogene^25,26,33,34^, but less is known about the metastatic mechanism of Ring1b in cancers. Our studies confirm that Ring1b promotes metastasis in breast cancers in vitro and in vivo. We further show that DDXs and EMT TFs contribute to site-selective recruitment of Ring1b complexes to the E-cadherin promoter. Complex 1 induces epithelial cells at an early hybrid EMT state by moderately inhibiting E-cadherin, and Complex 2 cooperates with Complex 1, which leads to E-cadherin silencing in mesenchymal-like cancer cells. Our conclusions may explain how breast cancer cells gradually lose the expression of E-cadherin and acquire metastatic characteristics by distinct Ring1b complexes. Our findings raise the possibility that targeting Ring1b complexes may provide a means of eliminating metastasis in breast cancer.

DDX3X and DDX5 are correlated with tumorigenesis in several cancers. For example, DDX5 mediates PDGF-induced EMT by displacing Axin from β-catenin in HT-29 cells^35^. DDX5 acts as a novel androgen receptor interacting protein to induce prostatic tumor development^36^. In breast cancer, DDX3X downregulates E-cadherin expression by binding its promoter, and is considered as a potential target for cancer treatment^15,37^. At present, the mechanism of DDXs function as transcription factors in cancers is unclear, and whether DDXs can recruit Ring1b in cancers has not been reported. While in non-tumor cells, DDX5 interacts with PRC1 to inhibit the reprogramming to pluripotency^38^. These reports raise the possibility that Ring1b may be involved in DDXs-dependent pathways in cancer. In our study, we verified the interaction between DDX3X/DDX5 and Ring1b, and confirmed the function and mechanism of these complexes in breast cancer metastasis.

Accumulating studies have shown that Snail and Twist bind to *E-box* region to silence E-cadherin expression in pancreatic and head and neck cancer^9,12,13,39^. Snail1 couples to different transcription factors to repress gene expression^40,41^. Twist2-coupled factors are still unclear. Here, we extend previous studies to further illustrate that EMT TFs can couple with Ring1b to mediate transcriptional suppression of E-cadherin in breast cancer.

No current studies address the competition between DDXs and EMT TFs on the E-cadherin promoter. Our data show that DDXs and EMT TFs bind on site-selective loci of the E-cadherin promoter, suggesting no obvious competition between Complex 1 and 2 in binding to E-cadherin promoter. Experiments in vitro and clinical evidence show that expression of DDXs positively correlates with EMT TFs, and high levels of DDXs and EMT TFs predict lower expression of E-cadherin, suggesting a cooperation of distinct Ring1b complexes in E-cadherin silencing.

Further studies have reported that Ring1b complexes exist in six distinct complexes (PRC1.1–PRC1.6) in mammals^42,43^. By CBX subunits, recruitment of PRC1.2 and PRC1.4 to chromodomain is via a PRC2-dependent mechanism^42,44,45^. Other types of PRC1 (so called noncanonical PRC1) directly bind transcription factors independently of PRC2 and H3K27me3^22,25,28,45^. Moreover, transcription factors seem to directly recruit PRC1 by binding Ring1b or other subunits^9,27,28,46^. In our studies, CBX4 and Ezh2 exist in both Complex 1 and 2, suggesting that the recruitment of Ring1b complexes on the E-cadherin promoter may be in a PRC2-dependent way. Whether DDXs or EMT TFs directly recruit PRC1 by Ring1b subunits is unclear. On the contrary, noncanonical PRC1 can induce E-cadherin expression in breast cell lines^47^. How noncanonical PRC1 participates in E-cadherin transcription in breast cancer is unknown.

Our clinical evidence shows that high expression of Ring1b with DDXs or EMT TFs predicted low levels of E-cadherin, metastatic behavior and poor prognosis. However, the expression of Ring1b have no obvious association with clinical stage, ER, PR, or HER-2 expression, and Ring1b show no distinct difference among breast cancer cell lines. Our interpretation of these results is that total Ring1b in cells could not exactly reflect and predict tumor volume and clinical stage in patients with breast cancer or connect with the metastatic ability in breast cancer cell lines. Nonetheless, the degree of metastasis in breast cancer cell lines still depends on the type of Ring1b complexes and the occupancy of Ring1b complexes or associated epigenetic markers in E-cadherin promoter.

## Materials and methods

### Cell culture

MCF-10A (10A), MDA-MB-231 (231), and MCF-7 cell lines were from American Type Culture Collection. HEK293T (293 T) cell lines were from the Cell Bank of the Chinese Academy of Sciences. All cell lines were characterized by DNA fingerprinting and isozyme detection. The 231 and 293T cells were cultured in DMEM supplemented with 10% fetal bovine serum (FBS) (Hyclone, USA). MCF-7 cells were maintained in RPMI-1640 medium supplemented with 10% FBS and 10 μg/ml human recombinant insulin (Sigma–Aldrich, Germany). The 10 A cells were cultured in DMEM/F12 with 5% horse serum (Gibco, USA), 0.02 μg/ml human EGF (Peprotech, USA), 0.5 μg/ml hydrocortisone (TCI, Japan), 0.1 μg/ml cholera toxin (Sigma–Aldrich) and 10 μg/ml insulin. All cell lines were grown at 37 °C under 5% CO_2_ in a humidified chamber.

### Antibodies

Primary antibodies were against Vimentin, Fibronectin, DDX3X, DDX5, Snail1 (sc-6260, sc-18825, sc-365768, sc-365164, sc-271977; Santa Cruz, USA), H2A (129418; GeneTex, USA), Ring1b, E-cadherin, H2AK119ub, Ezh2, H3K27me3, IgG, H3, HDAC1, H3K27ac, β-actin (#5694, #3195, #8240, #5246, #9733, #2729, #4620, #34589, #8173, #3700; Cell Signaling Technology, USA), HA, Flag (H9658, F1804; Sigma–Aldrich), IgG, CBX4 (AC011, A6221; ABclonal; China), and Twist2 (4173 R; Bioss, China).

### Tissue microarray and immunohistochemistry

Breast cancer tissue microarray chips contained 37 samples of invasive ductal carcinoma and matched adjacent breast tissue, and 87 samples of invasive ductal carcinoma and matched adjacent lymph node carcinoma tissue (BR804b, BR1005b, BR10010e; US. Biomax. Company, USA). All human tissues were collected under IRB and HIPPA approved protocols, and all samples were approved for commercial product development. Tumor staging was evaluated according to TNM classification of malignant tumors. Immunohistochemistry (IHC) staining was performed with the Super sensitive TM IHC Detection System Kit (Bioworld, USA). Sections were analyzed by Image-Pro Plus analysis software. An immunoreactive scoring system and mean integrated optical density (IOD) were used to evaluate protein expression.

### Plasmid and si-RNA transfections and lentiviral production

Recombinant plasmids were constructed in our laboratory and confirmed by DNA sequencing. pcDNA3.1(+)-HA-Ring1b, -Flag-DDX5, -Flag-Snail1 and -Flag-Twist2 were cloned between the *BamH* I and *EcoR* I sites; pcDNA3.1(+)-Flag-DDX3X was cloned between the *EcoR* I and *Xho* I sites; and pcDNA3.1(+)-HA-HDAC1 was cloned between the *BamH I* and *Xho I* sites. pLVTHM-sh-RNAs targeting Ring1b were 5-CAGTGAATTAATGTGCCCA-3 and 5-AACAATGCAGCAATGGCAA-3; targeting DDX3X were 5- AACAGGCAACAACTGTCCT-3 and 5-GCCGATATTGGTCAGCAGA-3, targeting DDX5 were 5-AGTGGAATCTTGATGAGCT-3 and 5-AGGTTCAGGTCGTTCCAGG-3. Control vector of pLVTHM-sh-RNAs targeted 5-AGTGAGATTCGTAGGATCT-3. pWPXLD-Ring1b, -DDX3X, -DDX5, -Flag-Snail1, -HA-Twist2, and -Ezh2 were constructed to stably overexpress relevant proteins. si-RNA sequences are as follow. si-control: UUCUUCGAACGUGUCACGUTT; si-Snail1^#^1: AACUCUGGAUUAGAGUCCUTT; si-Snail1^#^2: UUGAAGGCCUUUCGAGCCUTT; si-Twist2^#^1: UGUGUUCCUGAAUCUAGACTT; si-Twist2^#^2: AAACAAGCAACAUAUACACTT. Lipofectamine 2000 or 3000 was used for transfection (Invitrogen, USA). Lentivirus were packaged by cotransfection of respective constructs with second-generation packaging plasmids pMD2.G and psPAX2 into 293 T cells. The viral media were harvested and used to transduce cells accordingly. Expression was verified by western blot and FACS.

### Mice and metastatic models

Female BALB/c nude mice (5 weeks old) were obtained from Vital River Laboratory Animal Technology Co., Ltd (Beijing, China). All mice were housed in specific pathogen-free conditions. All animal procedures and studies were conducted in accordance with the Institutional Animal Care and Use Committee guidelines. For the metastatic models, GFP^+^ 231 cells (3 × 10^5^) resuspended in PBS (100 μl) were injected into the tail vein of mice. Six weeks after injection, lungs were harvested and used for sorting cancer cells and hematoxylin and eosin (H&E) staining.

### Western blots, co-immunoprecipitation (IP), and immunofluorescence

Cells were lysed in RIPA buffer in the presence of PMSF and protease inhibitor cocktail (#7012; Cell Signaling Technology). Protein concentrations were quantified by using BCA protein assay reagent kit (Bioworld, USA). For western blots, proteins were boiled with loading buffer and resolved by SDS-PAGE, then transferred onto nitrocellulose filter membranes (GE Healthcare, USA). After blocking, membranes were incubated with primary and secondary antibodies, and exposed to ECL reagent (ThermoFisher, USA). The detailed protein quantification of triplicates with error bars is displayed in Table.S2. For co-immunoprecipitation, cell extracts were incubated with antibodies and protein A/G magnetic beads (MCE, USA). After washing, precipitated proteins were analyzed by western blot. For immunofluorescence, cells were fixed in 4% paraformaldehyde and permeabilized with 0.1% TritonX-100 for 15 min, blocked with 5% FBS for 90 min, then incubated with primary and secondary antibodies. DAPI staining was performed before observation. Fluorescent images were captured using confocal microscope (FV1000; Olympus, Japan).

### Mass spectrometry (MS)

Lysates from cells were purified with anti-Ring1b antibody according to the method of co-immunoprecipitation. Fractions were resolved on SDS-PAGE, silver stained, and subjected to LC-MS/MS (Easy-nLC1000, Q Exactive; Thermo Fisher) sequencing and data analysis.

### Transwell migration and invasion assays

Cells were collected and resuspended in FBS-free DMEM. Twenty thousand cells were placed on 8-μm pore transwell filters (Corning, USA). For invasion assays, filters were coated with Matrigel (BD, UAS) in advance and for migration assays, this step was omitted. DMEM with 10% FBS was added to the bottom chamber as an attractant. Following incubation for 24–36 h, nonmigrated cells at the top of the filter were removed and cells at the bottom of the filters were fixed with 4% paraformaldehyde and stained with crystal violet. The total number of invaded cells in each chamber were quantified by counting at least four randomly chosen fields under ×20 magnification using a bright field microscope (IX71; Olympus).

### Cell cycle and MTT assays

Cells were grown in 10-cm plates to 50% confluency. For cell cycle assays, cells were collected and resuspended in 70% alcohol solution at −20 °C overnight. After adding RNase and propidium iodide, cells were analyzed by FACS (Canto II; BD); for MTT assays, cells were seeded in 48-well plates. After incubation for the indicated time, cells were treated with 0.05% MTT (M5655; Sigma–Aldrich, Germany) solution for 1 h. Then MTT was discarded and 0.2 mL DMSO was added. Absorbance was measured at 570 nm by a microplate reader (Syenergy; BioTek, USA).

### Reverse transcription, qRT-PCR, and qRT-PCR array

Total RNAs were extracted using TRIzol (Takara, China) reagent. Purified RNA (1 μg) was transcribed to complementary DNA using the GoScript reverse transcription system (Promega, USA). qRT-PCR was performed on a Roche Applied Science LightCycler 480 system using SYBR Green agent (Takara). Relative expression of mRNA was calculated using the 2^-ΔΔCt^ method. For qRT-PCR array analysis, the data from qRT-PCR were analyzed by Cluster 3.0 software, and the heatmap was exported by Tree View software. The sequence of primers referred to OriGene Technologies (Rockville, MD) are listed in supplementary information.

### Chromatin immunoprecipitation (ChIP)

Cells were grown in 10-cm plates to 80% confluency. SimpleChIP kits (#9003; Cell Signaling Technology) were used to obtain DNA fragments. Immunoprecipitated DNA fragments were detected by qRT-PCR using SimpleChIP Universal qPCR Master Mix regent (#88989; Cell Signaling Technology). Primer sets used to amplify the DNA fragment flanking region of E-cadherin promoters are listed in supplementary information.

### Statistical analysis

All preclinical assays were repeated three times. Results are presented as means ± standard error mean (SEM). Paired or unpaired *t*-test was performed using GraphPad Prism version 6 software. A value of *P* < 0.05 was considered to indicate a statistically significant difference and *P* ≥ 0.05 was considered as no significance (ns).

## Supplementary information

Supplementary Information

Supplementary Figure Legends

Figure S1

Figure S2

Figure S3

Figure S4

Figure S5

Figure S6

Figure S7

Figure S8

Figure S9

Figure S10

Table S1

Table S2

Reproducibility Checklist

Detailed Attribution of Authorship
