# Supplementary material for: Distinct Ring1b complexes defined by DEAD-box helicases and EMT transcription factors synergistically enhance E-cadherin silencing in breast cancer
Source: Cell Death Dis. 2021 Feb 19;12(2):202. doi: 10.1038/s41419-021-03491-4 (PMC7895950; doi:10.1038/s41419-021-03491-4)
Supplement: Supplementary file 1 — Supplementary Information [file 41419_2021_3491_MOESM1_ESM.docx]

**Supplementary Materials and Methods**

**Primer sequences for qRT-PCR:**

β-actin, forward 5-CACCATTGGCAATGAGCGGTTC-3, and reverse 5-AGGTCTTTGCGGATGTCCACGT-3;

CBX2, forward 5-GGAACATGAGAAGGAGGTGCAG-3, and reverse 5-GAAGAGGAGGAACTGCTGGACT -3;

CBX4, forward 5-GCTGCTGATCGCCTTCCAGAAC-3, and reverse 5-TTGGAACGACGGGCAAAGGTAG-3;

CBX6, forward 5-AGAGGGAGCGTGAGCTGTATGG-3, and reverse 5-GCTCGGCTTGACAGAGAAATGC-3;

CBX7, forward 5-AGGAAGAGAGGTCCGAAACCCA-3, and reverse 5-AGAAGCAGAGCTTCTCCTTGCC-3;

Ring1a, forward 5-CCTATCTGCCTGGACATGCTGA-3, and reverse 5-GCTTCTTTCGGCAGGTAGGACA-3;

Ring1b, forward 5-CAGTCACAGCATTGAGGAAGGAC-3, and reverse 5-GCTTCCTGATTGCTATGTGTGGA-3;

Pcgf1, forward 5-ACGAGACACAGCCACTGCTCAA-3, and reverse 5-TCCAAACCTCGGGACTGGTAGA-3;

Pcgf2, forward 5-CACTATCGTGGAGTGCCTGCAT-3, and reverse 5-GGTTTTATGGACCTGCACGTCAC-3;

Pcgf3, forward 5-AGGACAACGACTACCACCGCAG-3, and reverse 5-TCTTCAGATGCAAGACGGTCGC-3;

Pcgf4, forward 5-GGTACTTCATTGATGCCACAACC-3, and reverse 5-CTGGTCTTGTGAACTTGGACATC-3;

Pcgf5, forward 5-CAACAGTGACGGAATGCCTCCA-3, and reverse 5-TGTCTCATGAACTTGGTTGCCAC-3;

Pcgf6, forward 5-GTCACTTCTCGTTGAGGCTGGA-3, and reverse 5-AGGTATGAAGACATTCTGTGATGG-3;

Phc1, forward 5-TTCCTGCCAAGGCATCTCCAGT-3, and reverse 5-AGTGTAGGAGGCGGTACTGAAG-3;

Phc2, forward 5-TCAACGCAGGTTCCAGCACACT-3, and reverse 5-CGTCTCAGGCACACTTTTCTCC-3;

Phc3, forward 5-CCTACAAGTGCAACCACCAGCA-3, and reverse 5-TGCTGGAGACAAAGTGGGTGGT-3;

Yaf2, forward 5-CAGGTTACTCAGCAGTTTGTGCC-3, and reverse 5-CTGAGCACTACTCCGATCCACA-3;

Scmh1, forward 5-ACCGTCTTCAGCTTCCTCAAGC-3, and reverse 5-CACTACGAAGGTTGTGGCAGAG-3;

Scml1, forward 5-CACTAAGCACCCTTCAACCTGG-3, and reverse 5-GTCACTCGTGAGTAGGAGCAGA-3;

Scml2, forward 5-GCCAGCCTTTTAGTTCTTCCAGG-3, and reverse 5-TGCTGAGGAGATCGTTTGGTGC-3;

E2F6, forward 5-GCGGAGAGTGTATGACATCACC-3, and reverse 5-GTCAGAAAGTTCCTCCTGTAGCT-3;

KDM2B, forward 5-CATGGAGTGCTCCATCTGCAATG-3, and reverse 5-ACTTCGGACACTCCCAGCAGTT-3;

USP7, forward 5-GTCACGATGACGACCTGTCTGT-3, and reverse 5-GTAATCGCTCCACCAACTGCTG-3;

AUTS2, forward 5-CCTCCTCATCACAGCAACTTCC-3, and reverse 5-GAAGGCATTGCCACCAACTGCT-3;

E-cadherin, forward 5-GCCTCCTGAAAAGAGAGTGGAAG-3, and reverse 5-TGGCAGTGTCTCTCCAAATCCG-3;

Vimentin, forward 5-AGGCAAAGCAGGAGTCCACTGA-3, and reverse 5-ATCTGGCGTTCCAGGGACTCAT-3;

Fibronectin, forward 5-ACAACACCGAGGTGACTGAGAC-3, and reverse 5-GGACACAACGATGCTTCCTGAG-3;

N-cadherin, forward 5-CCTCCAGAGTTTACTGCCATGAC-3, and reverse 5-GTAGGATCTCCGCCACTGATTC-3;

ZEB1, forward 5-GGCATACACCTACTCAACTACGG-3, and reverse 5-TGGGCGGTGTAGAATCAGAGTC-3;

ZEB2, forward 5-AATGCACAGAGTGTGGCAAGGC-3, and reverse 5-CTGCTGATGTGCGAACTGTAGG-3;

Twist1, forward 5-GCCAGGTACATCGACTTCCTCT-3, and reverse 5-TCCATCCTCCAGACCGAGAAGG-3;

Twist2, forward 5-GCAAGATCCAGACGCTCAAGCT-3, and reverse 5-ACACGGAGAAGGCGTAGCTGAG-3;

Snail1, forward 5-TGCCCTCAAGATGCACATCCGA-3, and reverse 5-GGGACAGGAGAAGGGCTTCTC-3;

Snail2, forward 5-ATCTGCGGCAAGGCGTTTTCCA-3, and reverse 5-GAGCCCTCAGATTTGACCTGTC-3;

MMP2, forward 5-AGCGAGTGGATGCCGCCTTTAA-3, and reverse 5-CATTCCAGGCATCTGCGATGAG-3;

MMP9, forward 5-GCCACTACTGTGCCTTTGAGTC-3, and reverse 5-CCCTCAGAGAATVGCCAGTACT-3;

Cav2, forward 5-TTCTCTTTGCCACCCTCAGCTG-3, and reverse 5-GAAGCATCGTCCTACGCTCGTA-3;

Dsp, forward 5-CAACCATAGAGAAAGCAAACGCG-3, and reverse 5-TTTCTGTTGCCACTGCTGGGAC-3;

Fgfbp1, forward 5-TGGCAAACCAGAGGAAGACTGC-3, and reverse 5-GGAACCCGTTCTCTTTTGACCTC-3;

Krt19, forward 5-AGCTAGAGGTGAAGATCCGCGA-3, and reverse 5-GCAGGACAATCCTGGAGTTCTC-3;

Mst1r, forward 5-GTCAATGGGACTGAGTGTCTGC-3, and reverse 5-TCTCTGTACTGGAAGGTCCAGG-3;

Nudt13, forward 5-ATCACGCTGGTGTCAGATGGGA-3, and reverse 5-AACTTCTCGGCGGATGGTCTCT-3;

Occludin, forward 5-ATGGCAAAGTGAATGACAAGCGG-3, and reverse 5-CTGTAACGAGGCTGCCTGAAGT-3;

Rgs2, forward 5-CTCTACTCCTGGGAAGCCCAAA-3, and reverse 5-TTGCTGGCTAGCAGCTCGTCAA-3;

Spp1, forward 5-CGAGGTGATAGTGTGGTTTATGG-3, and reverse 5-GCACCATTCAACTCCTCGCTTTC-3;

Tspan13, forward 5-TCGCCATGTGCTCCAATCATAGG-3, and reverse 5-CTGTAGGTCAGCCAAACACCCA-3;

Ahnak, forward 5-CGTGAAGTCTTCAGCTCCTGCA-3, and reverse 5-GAGGTCTCCTTCCACTCCATCT-3;

Bmp1, forward 5-CCAATGGCTACTCTGCTCACATG-3, and reverse 5-AAGCCATCTCGGACCTCCACAT-3;

Cald1, forward 5-CTGTTCCTGCTGAAGGTGTACG-3, and reverse 5-CCTACCTTCAAGCCAGCAGTTTC-3;

Igfbp4, forward 5-ACCCACGAGGACCTCTACATCA-3, and reverse 5-CACACCAGCACTTGCCACGCT-3;

Itga5, forward 5-GCCGATTCACATCGCTCTCAAC-3, and reverse 5-GTCTTCTCCACAGTCCAGCAAG-3;

ItgaV, forward 5-AGGAGAAGGTGCCTACGAAGCT-3, and reverse 5-GCACAGGAAAGTCTTGCTAAGGC-3;

Moesin, forward 5-CTGATGGAGAGGCTGAAGCAGA-3, and reverse 5-ACGCTTCCGTTCCTGCTCAAGT-3;

Steap1, forward 5-GGCAATACTGGCTCTGTTGGCT-3, and reverse 5-GCGTGTATTGTGCCCAGTAGAAG-3;

Tcf4, forward 5-GCCTCTTCACAGTAGTGCCATG-3, and reverse 5-GCTGGTTTGGAGGAAGGATAGC-3;

Timp1, forward 5-GGAGAGTGTCTGCGGATACTTC-3, and reverse 5-GCAGGTAGTGATGTGCAAGAGTC-3;

Tmeff1, forward 5-CTCCAAGTGTGGACCCTGCAAA-3, and reverse 5-GGAACTCCCATCAGAAGCACAC-3;

Vcam1, forward 5-GATTCTGTGCCCACAGTAAGGC-3, and reverse 5-TGGTCACAGAGCCACCTTCTTG-3;

Wnt5a, forward 5-TACGAGAGTGCTCGCATCCTCA-3, and reverse 5-TGTCTTCAGGCTACATGAGCCG-3;

Wnt5b, forward 5-CAAGGAATGCCAGCACCAGTTC-3, and reverse 5-CGGCTGATGGCGTTGACCACG-3.

**Primer sequences for ChIP-qRT-PCR:**

Amplicon I, forward 5-ATGAAGCTCTACAGTTAA-3, and reverse 5- GCCAGGCTGGTCTGGAACTC-3;

Amplicon II, forward 5-CAACATGGTGAAACCCCGTCTG-3, and reverse 5-GTGAGCCATGAGCCACTGAGCT-3;

Amplicon III, forward 5-ACCTGAAATCCTAGCACTTTG-3, and reverse 5-CTCACTGCAGCCTCGAAC-3;

Amplicon IV, forward 5-CTGTGATCGCACCACTGCACT-3, and reverse 5-ACCCGGCCTCGCATAGACG-3;

Amplicon V, forward 5-CGTCTATGCGAGGCCGGGT-3, and reverse 5- TCCAAGGGCCCATGGCT-3;

Amplicon 1 (site 1), forward 5-CAACATGGTGAAACCCCGTCTG-3, and reverse 5-GTGAGCCATGAGCCACTGAGCT-3;

Amplicon 2 (site 2), forward 5-GCAGGTGAACCCTCAGC-3, and reverse 5-CACAGGTGCTTTGCAGTTCC-3.
