# Supplementary material for: Distinct Ring1b complexes defined by DEAD-box helicases and EMT transcription factors synergistically enhance E-cadherin silencing in breast cancer
Source: Cell Death Dis. 2021 Feb 19;12(2):202. doi: 10.1038/s41419-021-03491-4 (PMC7895950; doi:10.1038/s41419-021-03491-4)
Supplement: Supplementary file 2 — Supplementary Figure Legends [file 41419_2021_3491_MOESM2_ESM.docx]

**Fig. S1** **Metastatic role of** **Ring1b in breast cancer cells.** Ring1b was stably overexpressed or knocked down in cells using lentivirus. **A** Effect of TGF-β on PRC1-genes transcription. Total RNAs isolated from 10A, TGF-β-induced (15 ng/ml, 24 h) 10A and 231 cells were analyzed by qRT-PCR. **B** Effect of Ring1b on H2AK119ub expression. Total protein extraction was analyzed by western blot. **C** Effect of TGF-β and Ring1b on H2AK119ub expression. Total protein extraction from 231 and TGF-β-induced (15 ng/ml, 24 h) 231 cells was analyzed by western blot. **D, E** Function of Ring1b in cell migration and invasion. 231 cells were stimulated with TGF-β (15 ng/ml) for 24 h. Statistical analysis of cells migration and invasion by Transwell assays are presented in the bar graphs. Scale bar, 60 μm. **F** Immunofluorescent staining of Ring1b (red; TRITC) and E-cadherin (red; TRITC) in 231 cells. 231 cells were stimulated with TGF-β (15 ng/ml) for 24 h. Scale bar, 25 μm. **G, H** Schematic diagram of lung metastasis model of BALB/c mice by tail vein injection of 231 cells (3 × 10^5^). Quantification of mouse body weight every 3 weeks after tail vein injection. Error bars represent the means ± SEM. Unpaired *t*-test is performed to indicate a statistically significant difference. ns, P ≥ 0.05; *, P < 0.05; **, P < 0.01; ***, P < 0.001.

**Fig. S2 Effect of Ring1b on cell cycle and cell viability.** Ring1b was stably overexpressed or knocked down in cells using lentivirus. **A–D** Effect of Ring1b on cell cycle and cell viability in breast cell lines within 48 h. Cell cycle and viability were determined by FACS and MTT assays respectively.

**Fig. S3** **Verification of Ring1b-interacting proteins in 293T cells. A** SDS-PAGE and silver stained analysis of potential Ring1b interaction proteins in breast cell lines. Proteins were captured by IP and verified by LC-MS/MS. **B, C** HDAC1 interacts with DDX3X, DDX5, Snail1 and Twist2. The 293T cells were transfected with plasmids as indicated for 24 h. Proteins were captured by IP and analyzed by western blot. **D** Ezh2 interacts with DDX3X and DDX5. The 293T cells were transfected with pWPXLD-Ezh2, Flag-DDX3X and Flag-DDX5 for 24 h. Proteins were captured by IP and analyzed by western blot. **E** Ezh2 interacts with Snail1 and Twist2. The 293T cells were transfected with pWPXLD-Ezh2, Flag-Snail1 and HA-Twist2 for 24 h. Proteins were captured by IP and analyzed by western blot. **F** Ezh2 interacts with Ring1b and HDAC1. 293T cells were transfected with pWPXLD-Ezh2, HA-Ring1b and HA-HDAC1 for 24 h. Proteins were captured by IP and analyzed by western blot. **G** DDX3X does not interact with DDX5, Snail1 and Twist2. 293T cells were transfected with Flag-DDX3X, Flag-DDX5, Flag-Snail1 and HA-Twist2 for 24 h. Proteins were captured by IP and analyzed by western blot.

**Fig. S4 Ring1b complexes in breast cell lines. A–C** Ring1b complexes verified in breast cell lines. Lysates from 10A-Ring1b, MCF-7 and 231 cells were analyzed by IP followed by western blot with antibodies as indicated.

**Fig. S5 Ring1b-associated proteins silence E-cadherin expression *in vitro*.** Proteins as indicated were stably overexpressed or knocked down in cells using lentivirus or si-RNA. **A** Immunofluorescent staining of E-cadherin (green, FITC) and DAPI (blue) in transfected 10A cells. Scale bar, 50 μm. **B–F** Effect of Ring1b-associated proteins on E-cadherin expression. Total protein from 10A and 231 cells was analyzed by western blot. **G–J** Effect of Ring1b-associated proteins on E-cadherin transcription. Total RNAs isolated from 10A, and 231 cells were analyzed by qRT-PCR. **K, L** Effect of si-Snail1 or -Twist2 on E-cadherin expression. Total RNAs isolated from 231 cells were analyzed by qRT-PCR. Error bars represent the means ± SEM. Unpaired *t*-test is performed to indicate a statistically significant difference. *, P < 0.05; **, P < 0.01; ***, P < 0.001.

**Fig. S6 Ring1b-associated proteins promote invasion *in vitro*.** Proteins as indicated were stably overexpressed or knocked down in cells using lentivirus. **A–D** Effect of DDX3X, DDX5, Snail1and Twist2 on invasion of 10A cells. Statistical analysis for cells invasion are in bar graphs. Scale bar, 60 μm. **E–H** Effect of DDX3X, DDX5, Snail1and Twist2 on invasion of 231 cells. Statistical analysis of cells invasion are in bar graphs. Scale bar, 60 μm. Error bars represent the means ± SEM. Unpaired *t*-test is performed to indicate a statistically significant difference. ***, P < 0.001.

**Fig. S7 Expression of Ring1b-associated proteins in breast tissues.** **A** IHC staining of Ring1b, DDX3X, DDX5, Snail1 and Twist2 in breast normal and cancer tissues. Scale bar, 25 μm.

**Fig. S8 Ring1b-associated proteins are frequently up-regulated in breast invasive ductal carcinoma.** **A–E** Analysis of Ring1b, DDX3X, DDX5, Snail1and Twist2 expression in breast cancer and adjacent peritumor tissues (n = 37), and in metastatic and adjacent cancer tissues (Ring1b, n = 87; DDX3X, Twist2, n = 47; DDX5, Snail1, n = 44). IOD was used to evaluate protein expressions. Paired *t*-test is performed to indicate a statistically significant difference.

**Fig. S9 The correlation analysis of DDXs and EMT TFs in breast cancer tissues. A** Double factor analysis of E-cadherin expression in cancer tissues. E-cadherin expression was determined by immunoreactive scoring system. IOD was used to evaluate protein expressions. Paired *t*-test is performed to indicate a statistically significant difference.

**Fig. S10 The survival probability of patients with breast cancer. A** Single factor analysis of survival curves in cancer tissues. **B** Multiple factor analysis of survival curves in cancer tissues. The data from TCGA atlas were analyzed by Kaplan-Meier method.
