# Supplementary figures and images for: Distinct Ring1b complexes defined by DEAD-box helicases and EMT transcription factors synergistically enhance E-cadherin silencing in breast cancer
Source: Cell Death Dis. 2021 Feb 19;12(2):202. doi: 10.1038/s41419-021-03491-4 (PMC7895950; doi:10.1038/s41419-021-03491-4)

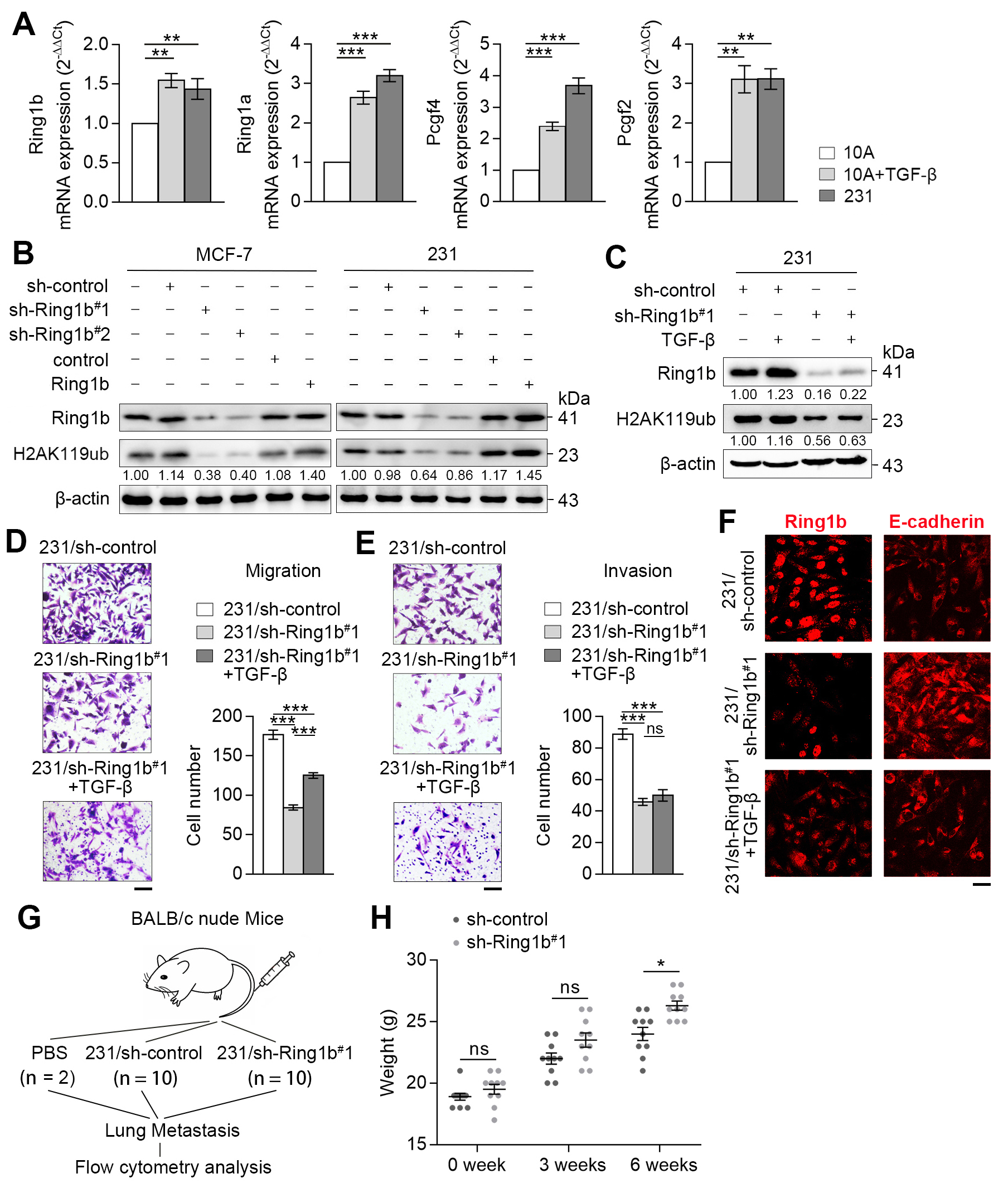

Supplement: Supplementary file 3 — Figure S1 [file 41419_2021_3491_MOESM3_ESM.tif]

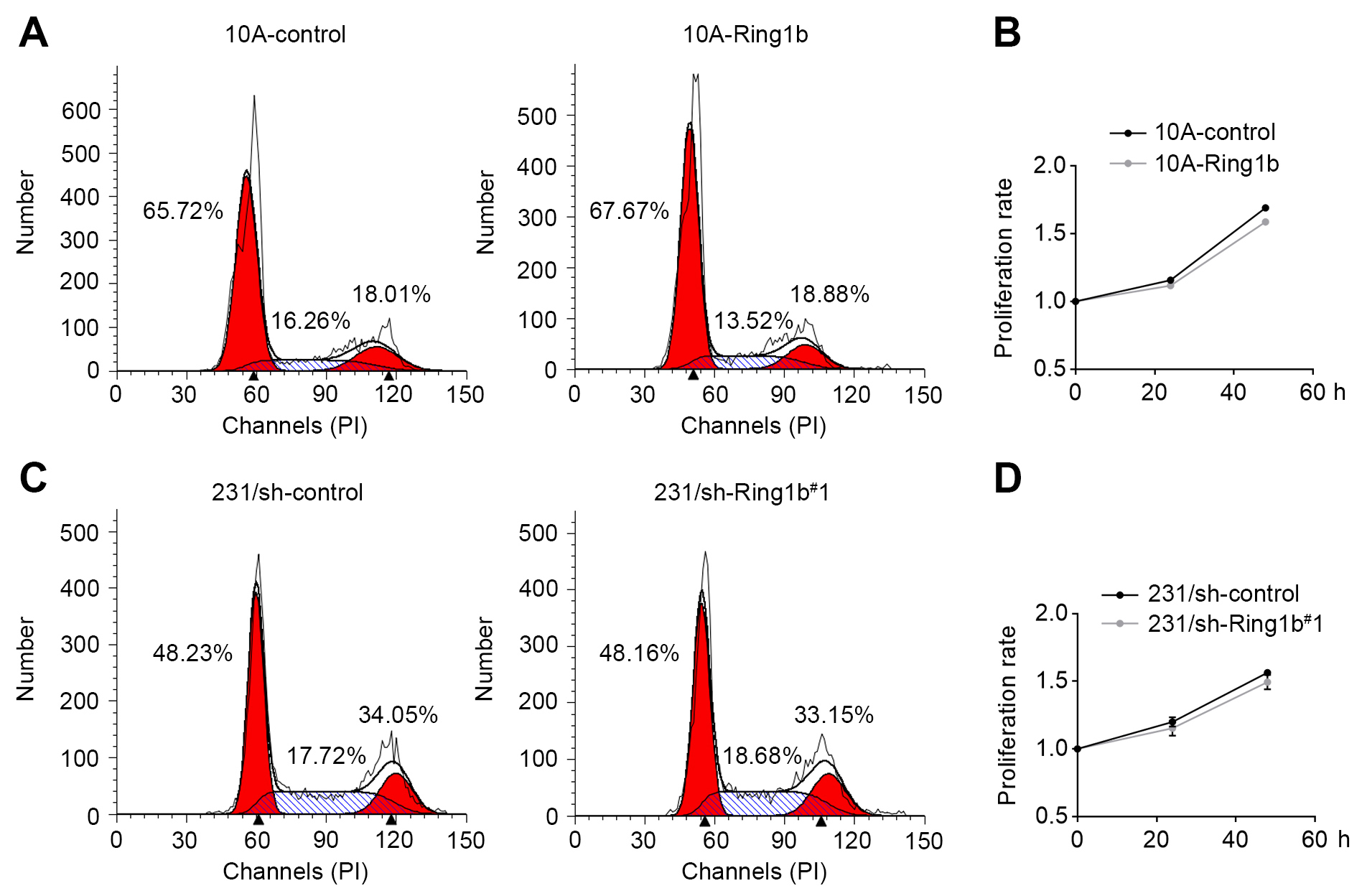

Supplement: Supplementary file 4 — Figure S2 [file 41419_2021_3491_MOESM4_ESM.tif]

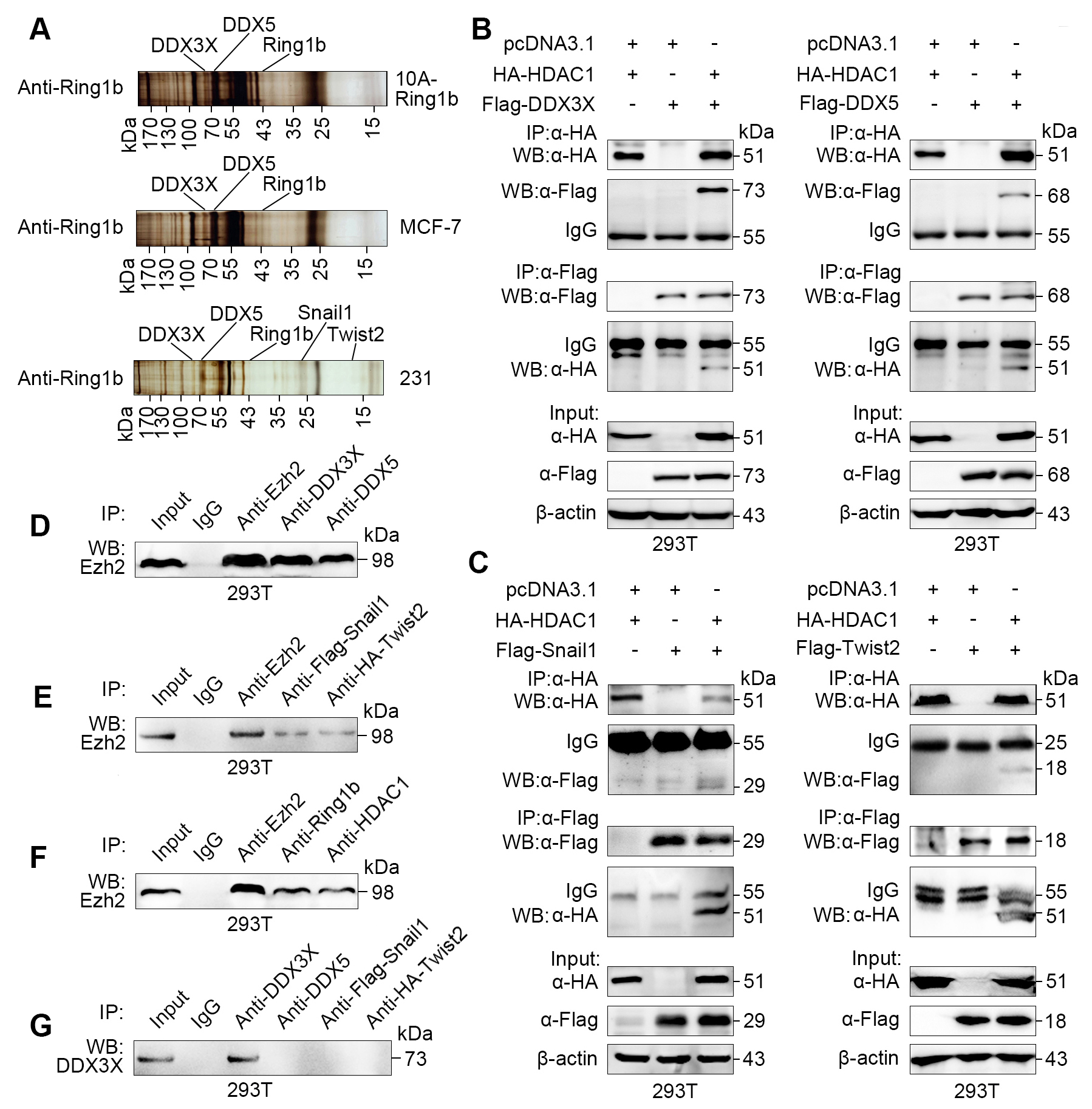

Supplement: Supplementary file 5 — Figure S3 [file 41419_2021_3491_MOESM5_ESM.tif]

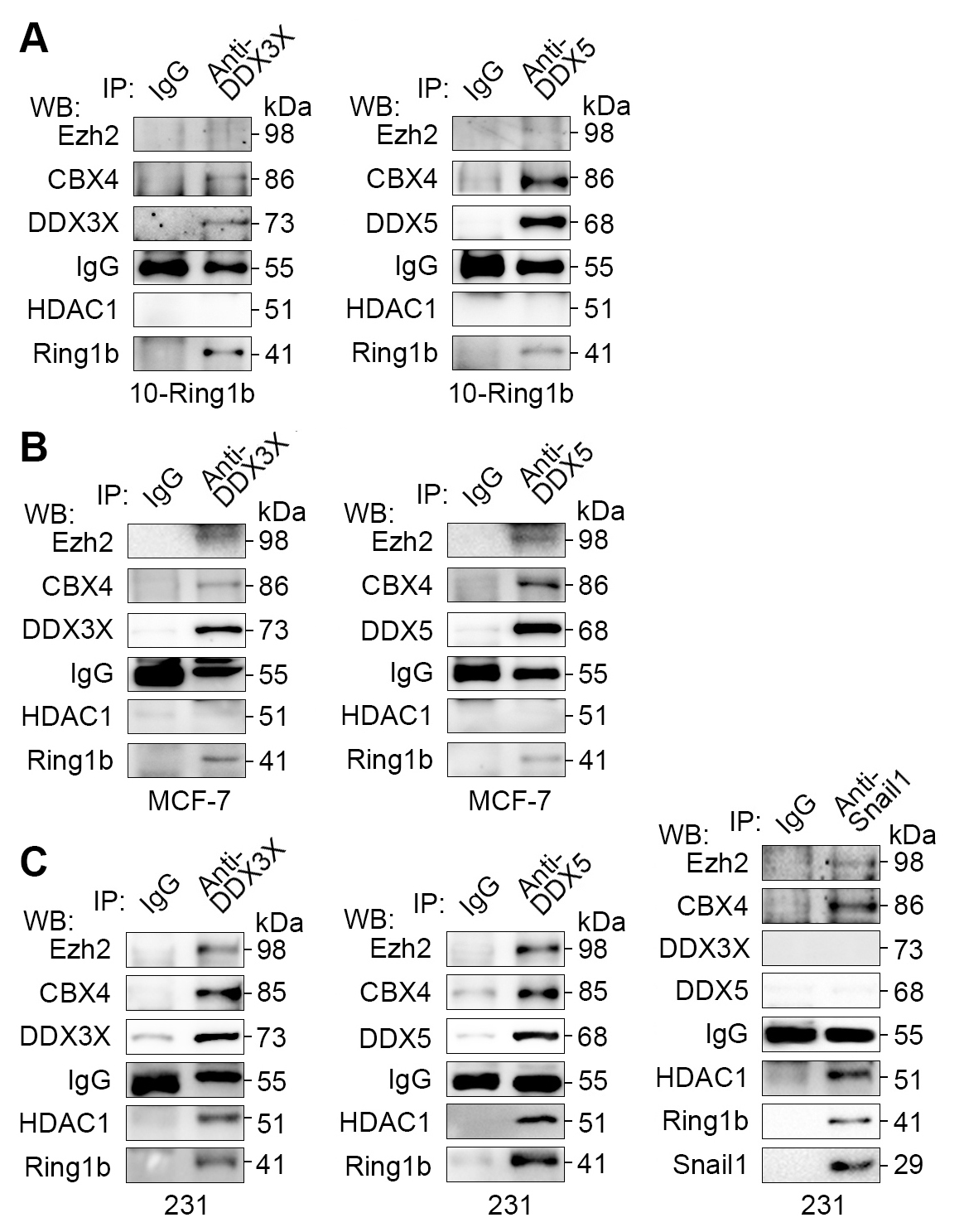

Supplement: Supplementary file 6 — Figure S4 [file 41419_2021_3491_MOESM6_ESM.tif]

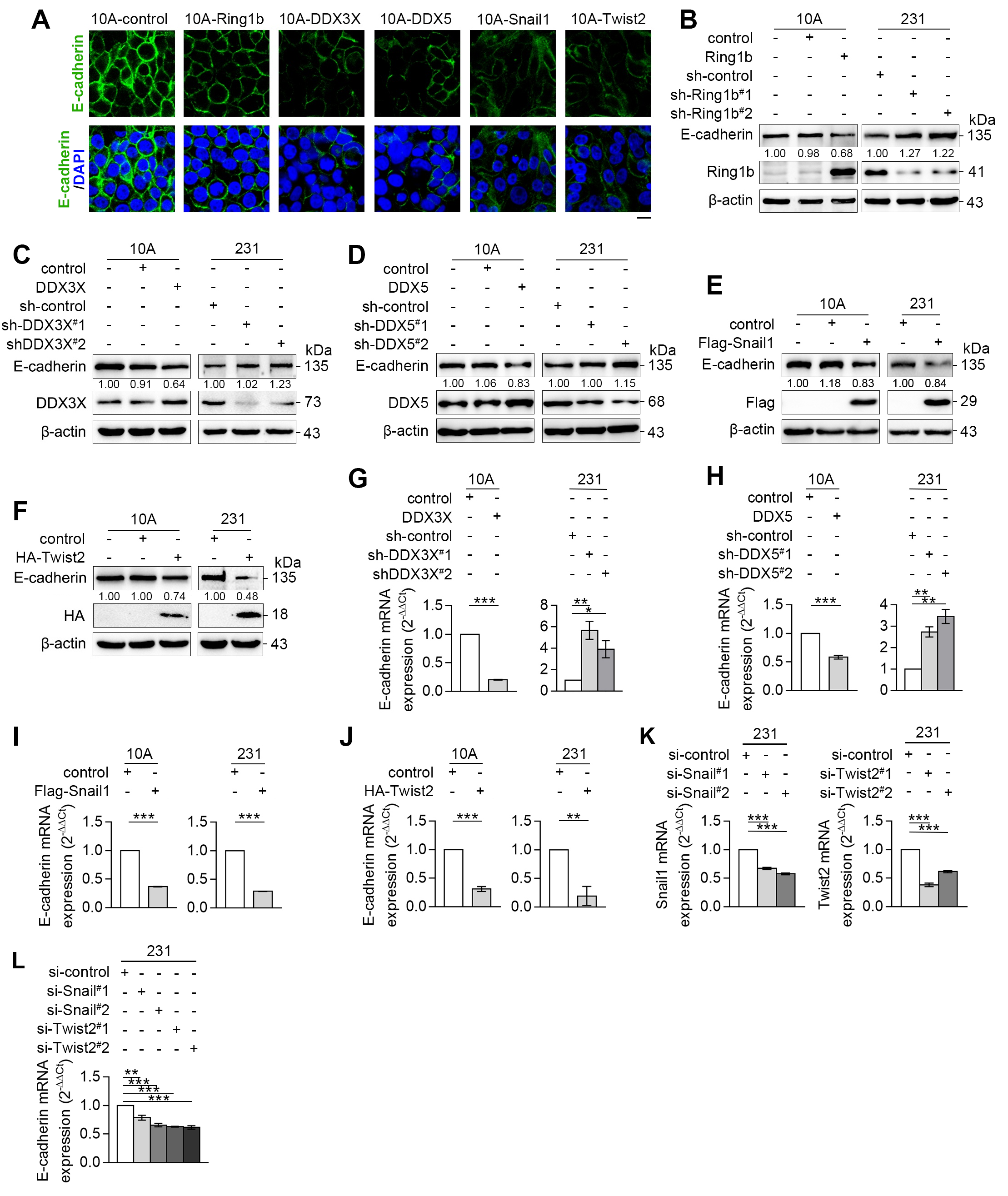

Supplement: Supplementary file 7 — Figure S5 [file 41419_2021_3491_MOESM7_ESM.tif]

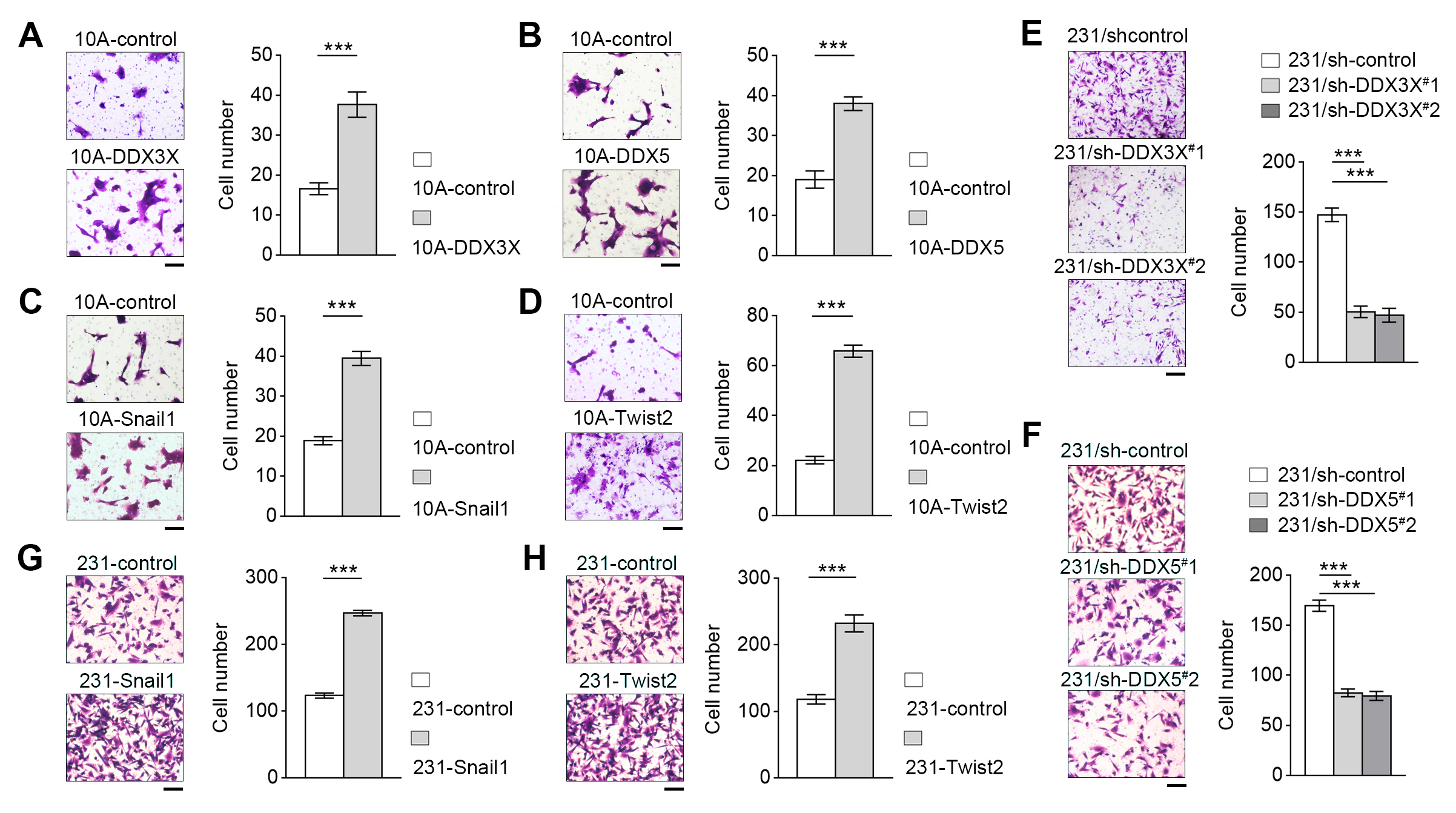

Supplement: Supplementary file 8 — Figure S6 [file 41419_2021_3491_MOESM8_ESM.tif]

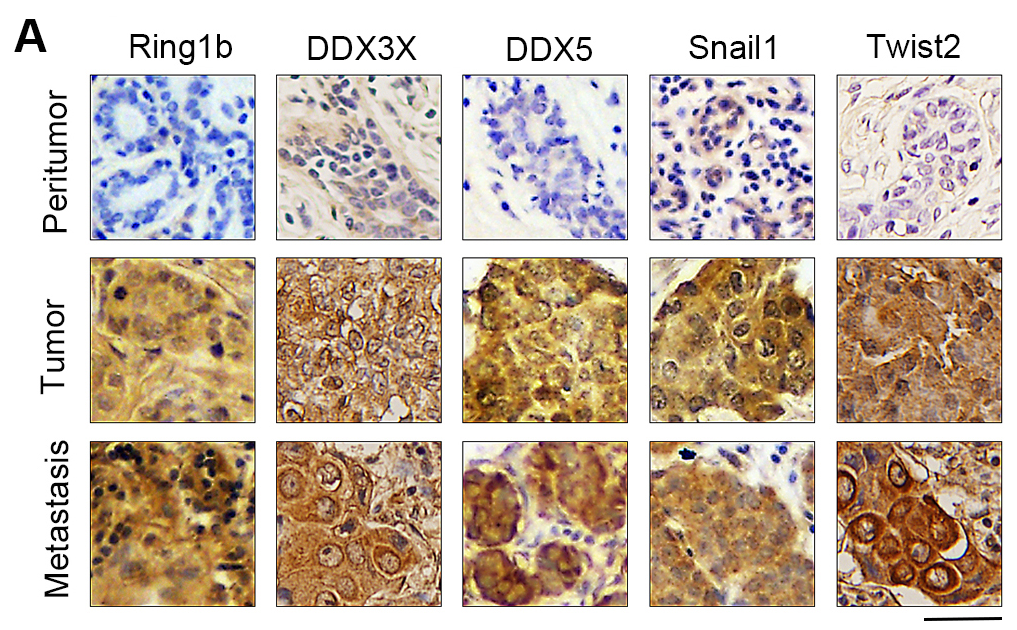

Supplement: Supplementary file 9 — Figure S7 [file 41419_2021_3491_MOESM9_ESM.tif]

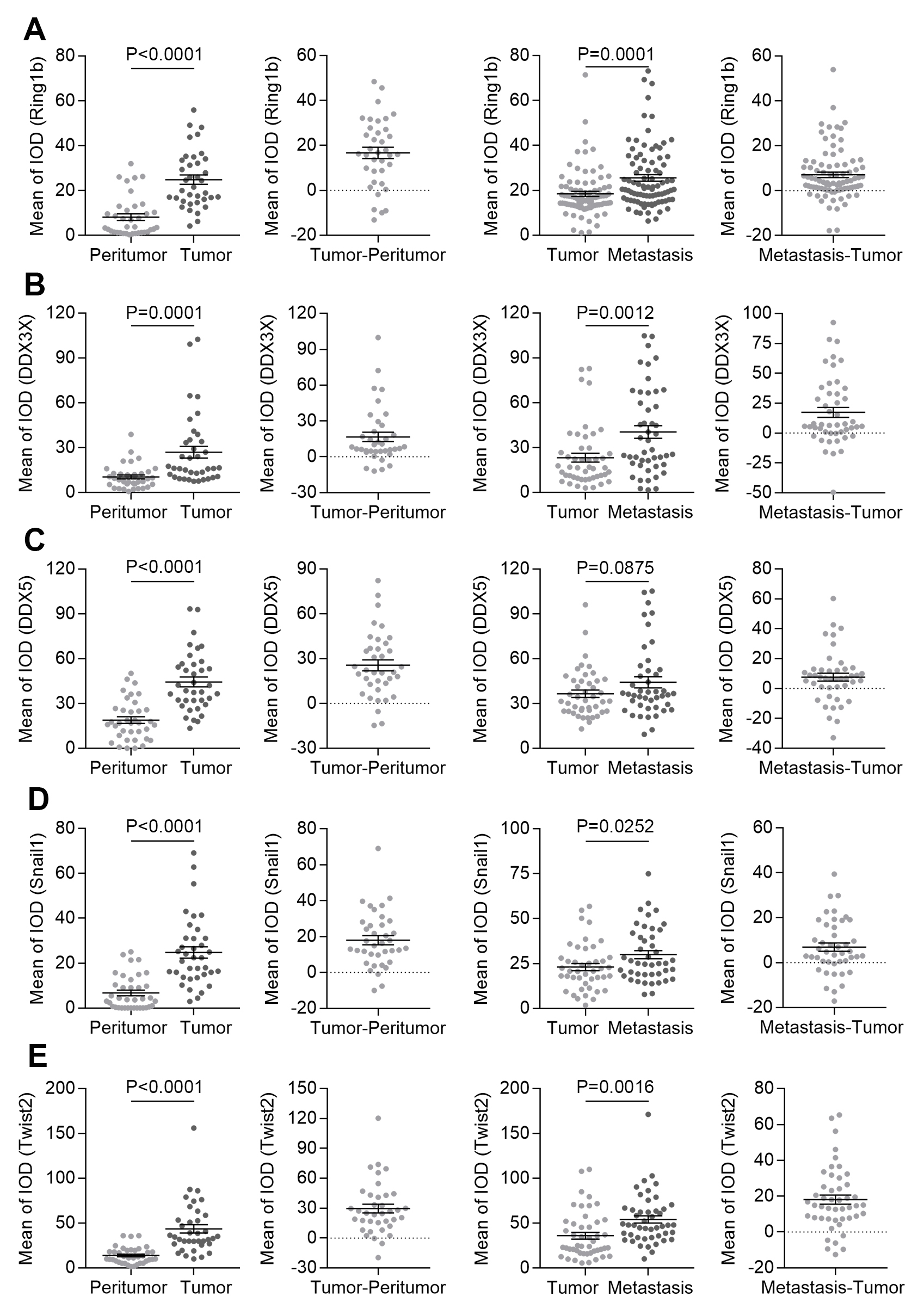

Supplement: Supplementary file 10 — Figure S8 [file 41419_2021_3491_MOESM10_ESM.tif]

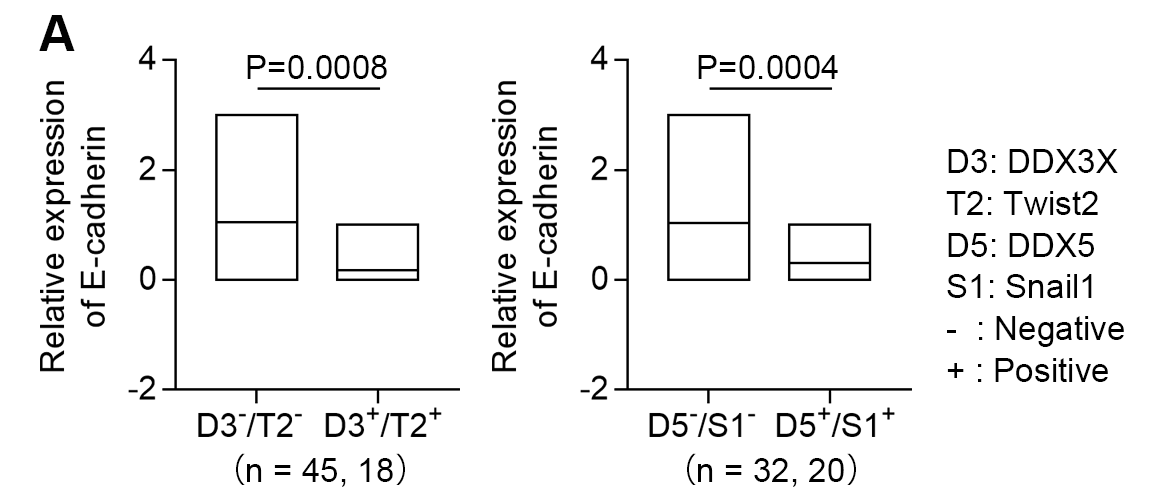

Supplement: Supplementary file 11 — Figure S9 [file 41419_2021_3491_MOESM11_ESM.tif]

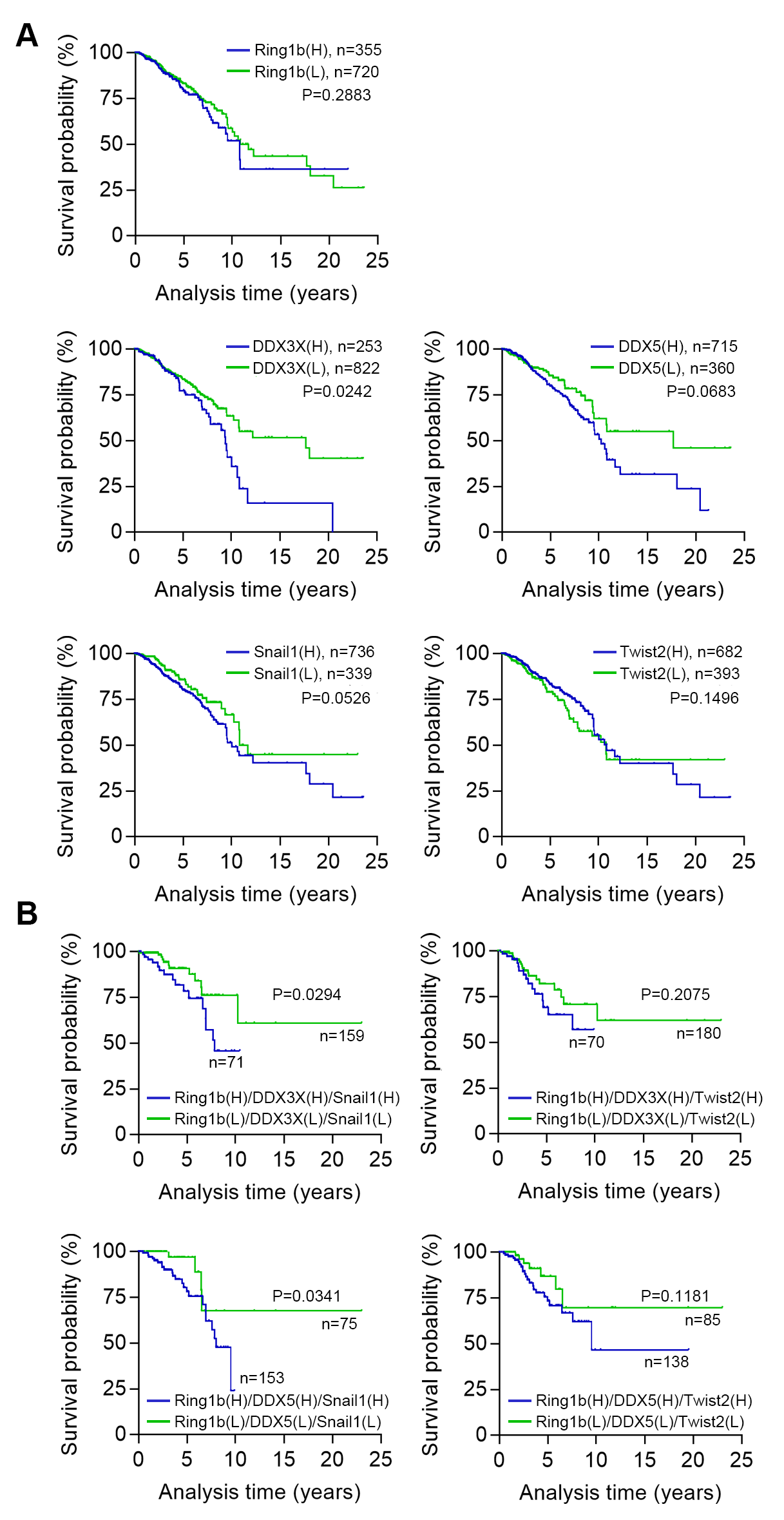

Supplement: Supplementary file 12 — Figure S10 [file 41419_2021_3491_MOESM12_ESM.tif]
