# Supplementary material for: Distinct Ring1b complexes defined by DEAD-box helicases and EMT transcription factors synergistically enhance E-cadherin silencing in breast cancer
Source: Cell Death Dis. 2021 Feb 19;12(2):202. doi: 10.1038/s41419-021-03491-4 (PMC7895950; doi:10.1038/s41419-021-03491-4)
Supplement: Supplementary file 16 — Detailed Attribution of Authorship [file 41419_2021_3491_MOESM16_ESM.pdf]

DECLARATION OF CONTRIBUTIONS TO ARTICLE

ADMC

Manuscript Number:	Journal Name:	
CDDIS-20-3506-R	*Cell Death & Disease*	(the 'Journal')

Proposed Title of the Contribution:

Distinct Ring1b complexes defined by DEAD-box helicases and EMT transcription factors synergistically enhance E-cadherin silencing in breast cancer (the 'Contribution)

Author(s):

Yawei Wang, Yingying Sun, Chao Shang, Lili Chen, Hongyu Chen, Dake Wang and Xianlu Zeng (the 'Authors')

For all *CDDis* articles, each person named as an author in the published version must be able to show he or she has contributed substantially to the article.

Authorship credit should be based on 1) substantial contributions to conception and design, acquisition of data, or analysis and interpretation of data; 2) drafting the article or revising it critically for important intellectual content; and 3) final approval of the version to be published. Authors should meet conditions 1, 2 and 3.

Any person who cannot be shown to have made a substantial contribution to the article cannot be listed as an author in the final version. The name of any person who is deemed to have made a minor contribution can, however, appear in the Acknowledgments section of the article.

Please complete the table below to indicate the contributions of all named authors to the manuscript.

Author Full Name:	Specification of Contribution to the Manuscript:
Yawei Wang	1，2，3.
Yingying Sun	1，2，3.
Chao Shang	1，2，3.
Lili Chen	1，2，3.
Hongyu Chen	1，2，3.
Dake Wang	1，2，3.
Xianlu Zeng	1，2，3.

Please complete the table below to indicate the contributions of all named authors to the figures.

Figure 1:
Yawei Wang, Yingying Sun, Chao Shang, Lili Chen and Xianlu Zeng

Figure 2:
Yawei Wang, Lili Chen, Hongyu Chen, Dake Wang and Xianlu Zeng

Figure 3:
Yawei Wang, Yingying Sun, Chao Shang, Lili Chen and Xianlu Zeng

Figure 4:
Yawei Wang, Yingying Sun, Chao Shang, Lili Chen and Xianlu Zeng

Figure 5:
Yawei Wang, Yingying Sun, Chao Shang, Lili Chen, Hongyu Chen, Dake Wang and Xianlu Zeng

Figure 6:
Yawei Wang, Hongyu Chen, Dake Wang and Xianlu Zeng

Signed for and on behalf of the Author(s):	Print Name:	Date:
曾宪录	Xianlu Zeng	27/11/2020
